# TMEM16F Regulates Spinal Microglial Function in Neuropathic Pain States

**DOI:** 10.1016/j.celrep.2016.05.039

**Published:** 2016-06-21

**Authors:** Laura Batti, Mayya Sundukova, Emanuele Murana, Sofia Pimpinella, Fernanda De Castro Reis, Francesca Pagani, Hong Wang, Eloisa Pellegrino, Emerald Perlas, Silvia Di Angelantonio, Davide Ragozzino, Paul A. Heppenstall

**Affiliations:** 1 EMBL Mouse Biology Unit, Via Ramarini 32, Monterotondo 00015, Italy; 2 Molecular Medicine Partnership Unit (MMPU), 69117 Heidelberg, Germany; 3 Center for Life Nanoscience, Istituto Italiano di Tecnologia, Viale Regina Elena 291, 00161 Rome, Italy; 4 Istituto Pasteur-Fondazione Cenci Bolognetti and Department of Physiology and Pharmacology, Sapienza University of Rome, Piazzale Aldo Moro, 5 00185 Rome, Italy; 5 Pharmacology Institute, University of Heidelberg, Im Neuenheimer Feld 366, 69120 Heidelberg; 6 IRCCS Neuromed, Via Atinese, Pozzilli 86077, Italy

## Abstract

Neuropathic pain is a widespread chronic pain state that results from injury to the nervous system. Spinal microglia play a causative role in the pathogenesis of neuropathic pain through secretion of growth factors and cytokines. Here, we investigated the contribution of TMEM16F, a protein that functions as a Ca^2+^-dependent ion channel and a phospholipid scramblase, to microglial activity during neuropathic pain. We demonstrate that mice with a conditional ablation of TMEM16F in microglia do not develop mechanical hypersensitivity upon nerve injury. In the absence of TMEM16F, microglia display deficits in process motility and phagocytosis. Moreover, loss of GABA immunoreactivity upon injury is spared in TMEM16F conditional knockout mice. Collectively, these data indicate that TMEM16F is an essential component of the microglial response to injury and suggest the importance of microglial phagocytosis in the pathogenesis of neuropathic pain.

## Highlights

• Microglial TMEM16F channels are required for neuropathic pain development in mice
• TMEM16F-deficient microglia display deficits in process motility and phagocytosis
• Deleting TMEM16F spares injury-induced loss of spinal cord GABA immunoreactivity
• Microglial phagocytosis may contribute to neuropathic pain development

## Graphical Abstract

**Figure:**
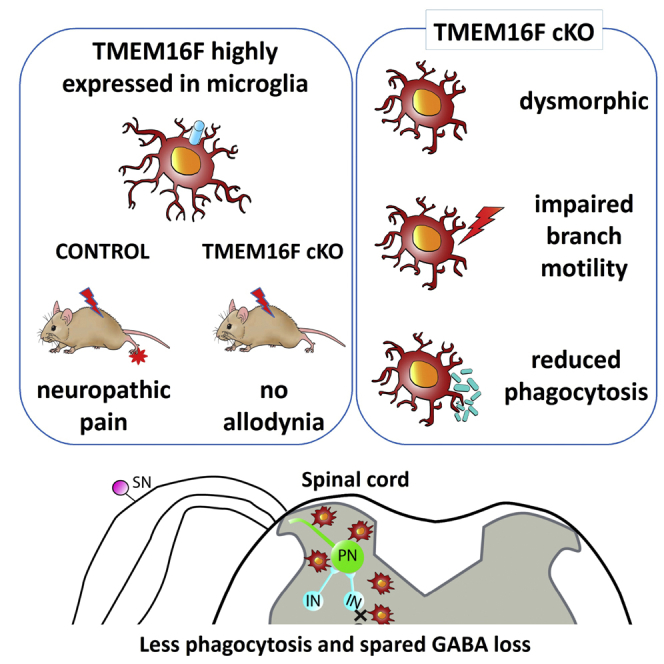

## Teaser

Batti et al. suggest that microglial phagocytosis may contribute to development of neuropathic pain. Mice with conditional TMEM16F ablation in microglia do not develop mechanical pain upon nerve injury. The authors show that TMEM16F-deficient microglia display deficits in process motility and phagocytosis.

## Introduction

Neuropathic pain is a widespread and debilitating clinical condition that is triggered by a lesion in the nervous system. (Campbell and Meyer, 2006, Costigan et al., 2009). It is becoming increasingly apparent that spinal microglia play a causative role in the pathogenesis of neuropathic pain (Scholz and Woolf, 2007). Peripheral nerve injury is associated with a pronounced recruitment of microglia to the spinal cord, and a conversion of these cells into a reactive state (Gehrmann et al., 1991), whereby they increase synthesis and release of bioactive molecules (Clark et al., 2007, Clark et al., 2013).

The most characterized mechanism through which microglia contribute to neuropathic pain involves a molecular pathway that is dependent upon upregulation of microglial purinergic P2X4 receptors (Beggs et al., 2012, Tsuda et al., 2003) and increased release of brain-derived neurotrophic factor (BDNF) from microglia, which acts on dorsal horn lamina I neurons to shift their transmembrane anion gradient (Coull et al., 2003, Coull et al., 2005). As a result, inhibitory synaptic signaling through GABA_A_ and glycine receptors is diminished. Furthermore, loss of inhibition is exacerbated by reduced production and release of spinal GABA (Lever et al., 2003, Moore et al., 2002) and injury-induced loss of GABAergic interneurons in the dorsal horn (Scholz et al., 2005, Sugimoto et al., 1990).

Microglia also have a well-established role in the detection and removal of apoptotic neuronal material (Davalos et al., 2005, Fu et al., 2014, Sierra et al., 2013). Upon injury, activated microglia converge on the dorsal horn in response to chemokine and ATP signaling and survey and modify sensory afferent input for damage via probing and extension of their processes (Davalos et al., 2005). Critical to this function is the activation of ion channels, which, through modulation of membrane potential, cell volume, and ion concentration promotes the movement of microglial processes and the initiation of phagocytosis. However, it is not known whether these mechanisms are important for aberrant nociceptive processing under pathological pain conditions.

Recently, a family of ion channels has been identified belonging to the TMEM16 family of proteins, which display functional diversity and may contribute to microglial function. The founding member of this family TMEM16A acts as a Ca^2+^-activated Cl^−^ channel (Caputo et al., 2008, Schroeder et al., 2008, Yang et al., 2008), and another member TMEM16F has been proposed to be a Ca^2+^-dependent phospholipid scramblase (Suzuki et al., 2010), and a Ca^2+^-activated channel with either anion (Almaça et al., 2009, Martins et al., 2011, Tian et al., 2012) or non-selective cation (Yang et al., 2012) permeability. Each of these molecular mechanisms could contribute to spinal microglial function in neuropathic pain states.

To investigate whether TMEM16 channels play a role in neuropathic pain, we assayed the expression levels of all TMEM16 family members in microglia and observed high enrichment of TMEM16F transcript. We thus generated a conditional knockout mouse line in which TMEM16F is genetically ablated in cells of the myeloid lineage. We demonstrate that, in the absence of TMEM16F, microglia are dysmorphic and exhibit deficits in process motility and phagocytosis. Moreover, TMEM16F conditional knockout mice display a pronounced reduction in mechanical hypersensitivity after peripheral nerve injury, suggesting that the classical scavenger function of microglia could be a factor in the pathogenesis of neuropathic pain.

## Results

### TMEM16 Expression Profiling and Generation of a TMEM16F Conditional Knockout Mouse Line

**Figure 1:**
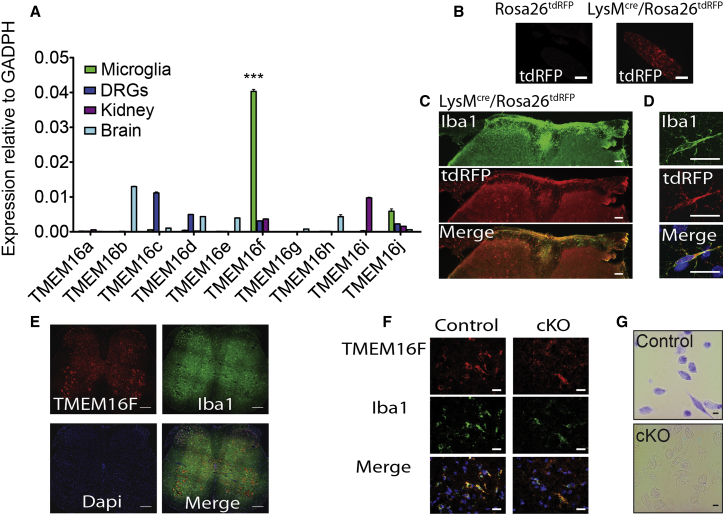
TMEM16 Expression and Generation of Conditional TMEM16F Knockout Mice (A) RT-PCR analysis of TMEM16 transcripts in microglia, dorsal root ganglia (DRGs), kidney, and brain; p < 0.0001; n = 3. (B) Representative images of injured sciatic nerve from indicated genotypes, 7 days after injury. (C) Iba1 (green) and RFP (red) immunofluorescence in the lumbar spinal cord *LysM*^*Cre*^*/Rosa26*^*tdRFP*^ mouse, 3 days after injury. (D) Iba1 (green) and RFP (red) immunofluorescence of microglia in the hippocampus from *LysM*^*Cre*^*/Rosa26*^*tdRFP*^ mouse. (E) In situ hybridization for TMEM16F (red) and Iba1 (green) immunofluorescence of microglia in spinal cord, 7 days after injury. (F) High-magnification representative images of TMEM16F in situ hybridization in control and cKO mice. (G) In situ hybridization for TMEM16F in peritoneal macrophages cells from control and cKO mice. Values are mean ± SEM. Scale bars, 100 and 40 μm (B), 100 μm (C), 20 μm (D, F, and G), 300 μm (E). See also Figure S1.

In order to identify TMEM16 proteins with a potential role in pain processing, we performed expression profiling of all ten TMEM16 family members in dorsal root ganglia (DRG), microglia, and brain and compared their relative expression to basal levels in kidney using qRT-PCR. We observed a strikingly high expression of TMEM16F in microglia that was not apparent in other tissues tested (Figure 1A). Moreover, TMEM16F is the predominant microglial TMEM16 transcript with levels 4-fold higher than the next highest transcript TMEM16J.

To investigate the significance of the high expression of TMEM16F in microglia, we generated conditional TMEM16F knockout mice (Figure S1A). We tested two separate Cre driver lines, *CX3CR1*^*Cre*^ (Yona et al., 2013) and *LysM*^*Cre*^ (Clausen et al., 1999), crossed with Cre-dependent reporter lines for selective microglial and macrophage recombination. As previously reported (Eriksson et al., 1993), we observed substantial microgliosis in ipsilateral spinal cord upon injury. However, in *CX3CR1*^*Cre*^*::Rosa26*^*tdRFP*^ mice, reporter expression was evident in both microglia and neurons throughout the spinal cord (Figure S1E). In contrast, *LysM*^*Cre*^*::Rosa26*^*tdRFP*^ mice displayed selective expression in macrophages recruited to the injury site, and in microglia in the spinal cord and brain (Figures 1B–1D). In situ hybridization for TMEM16F and immunostaining for microglial marker Iba1 in spinal cord sections from injured mice, confirmed robust TMEM16F expression in spinal microglia from *LysM*^*Cre*^*::TMEM16F*^*fl/+*^ mice and a decrease in TMEM16F mRNA in microglia and macrophages from *LysM*^*Cre*^*::TMEM16F*^*fl/fl*^ mice (Figures 1E–1G). We thus performed all further analysis on the *LysM*^*Cre*^*:: TMEM16F*^*fl/fl*^ (cKO) line using *TMEM16F*^*fl/fl*^ (in the absence of Cre) or *LysM*^*Cre*^*:: TMEM16F*^*fll+*^ mice as controls.

### Microglial TMEM16F Is Required for Neuropathic Pain Development

**Figure 2:**
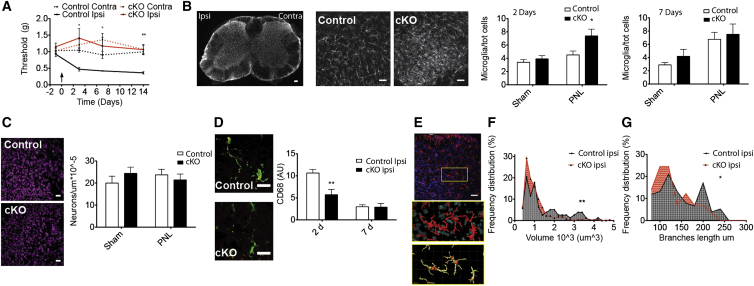
Microglial TMEM16F Is Required for the Development of Mechanical Hypersensitivity in the PNL Neuropathic Pain Model (A) Paw withdrawal thresholds of *TMEM16F*^*fl/fl*^ (control) and *LysM*^*cre*^*/TMEM16F*^*fl/fl*^*(cKO)* mice showing ipsilateral (ipsi) and contralateral (contra) paw withdrawals before and after partial nerve ligation (PNL). n = 9; p < 0.05. (B) From left to right: microglial Iba1 immunofluorescence in the lumbar spinal cord after injury (left side), magnified images from control and cKO tissues 2 days after PNL. Bar graphs show microglial densities in injured (PNL) and non-injured (sham) tissue 2 (left) and 7 (right) days after injury. n = 8/9; p < 0.05. (C) Neuronal marker NeuN immunofluorescence in the ipsilateral dorsal horn from control (top) and cKO (bottom) mice; bar graph showing neuronal density in the imaged volume. n = 10/7; p < 0.05. (D) CD68 (red) and Iba1 (green) immunofluorescence in the ipsilateral dorsal horn from control and cKO mice. CD68 immunoreactivity in Iba1 positive cells at 2 and 7 days after injury. n = 6; p < 0.01. (E) Top: Iba1 (red) and DAPI (blue) immunolabeling in the ipsilateral dorsal horn; 3D image segmentation using IMARIS Bitplane surface (middle) and filament (bottom) algorithms. (F) Frequency distribution of microglial volume in the ipsilateral dorsal horn from control and cKO mice, n = 79/277 cells; p = 0.005. (G) Frequency distribution of microglial total branch length in the ipsilateral dorsal horn from control and cKO mice; p = 0.0293. Values are mean ± SEM. Scale bars represent 100 μm (B), 30 μm (C), 20 μm (D), 40 μm, and 7 μm (E). See also Figures S2 and S3.

To determine whether TMEM16F contributes to microglial function in pain states, we monitored nociceptive behavior in *LysM*^*Cre*^*::TMEM16F*^*fl/fl*^ mice in models of neuropathic and inflammatory pain. In control mice subjected to partial nerve ligation (PNL), we observed a pronounced mechanical allodynia that peaked at 7 days post-injury. Strikingly, deletion of TMEM16F in microglia and macrophages prevented the development of allodynia, and mechanical withdrawal thresholds remained at pre-injury levels throughout the monitoring period (Figure 2A), an effect that was observed in both male and female mice (Figure S2A).

To investigate the contribution of microglial TMEM16F to inflammatory pain, we used the Complete Freund’s Adjuvant (CFA) model. We observed mechanical allodynia 48 hr after intraplantar injection of CFA in control animals. In contrast to the PNL model, *LysM*^*Cre*^*::TMEM16F*^*fl/fl*^ mice developed mechanical hypersensitivity to the same extent as litter mate controls (Figure S2B).

To explore whether TMEM16F contributes to proliferative, migratory, and phagocytic responses of microglia upon peripheral nerve injury, we performed quantitative image analysis on immunohistologically labeled spinal cord sections. Intriguingly, the total number of Iba1 positive microglia increased more rapidly after injury in lumbar spinal cords from *LysM*^*Cre*^*::TMEM16F*^*fl/fl*^ compared to control mice, and 3 days post-injury this had already reached maximal levels. In contrast, microglial density from control mice required 7 days to reach similar levels (Figure 2B). We observed no difference in the number of neuronal cell bodies in at seven days post-injury between control and conditional knockout (cKO) animals (Figure 2C).

We investigated the activation status of microglia by quantifying lysosomal protein CD68 immunoreactivity (Holness and Simmons, 1993) in Iba1 positive cells and observed a dramatic increase at 2 days post-injury in control spinal cords that had normalized to baseline levels 7 days post-injury. In contrast, in spinal cords from *LysM*^*Cre*^*::TMEM16F*^*fl/fl*^ mice, CD68 immunoreactivity was significantly reduced compared to control (Figure 2D), suggesting that microglial phagocytic activity was impaired in the absence of TMEM16F.

We further examined morphological characteristics of spinal microglia, using 3D reconstruction of spinal cord confocal stacks and segmentation of Iba1 positive cells (Figure 2E). Microglia were significantly smaller in size and less branched in the ipsilateral dorsal horn of cKO mice compared to controls (Figures 2F and 2G).

As an additional measure of microglial function, we performed immunostaining for P2X4 receptors. Upon PNL, there was an increase in P2X4 staining in the ipsilateral side compared to contralateral (73% ± 16%), also observed in the cKO mice (92% ± 17%; Figure S3).

**Figure 3:**
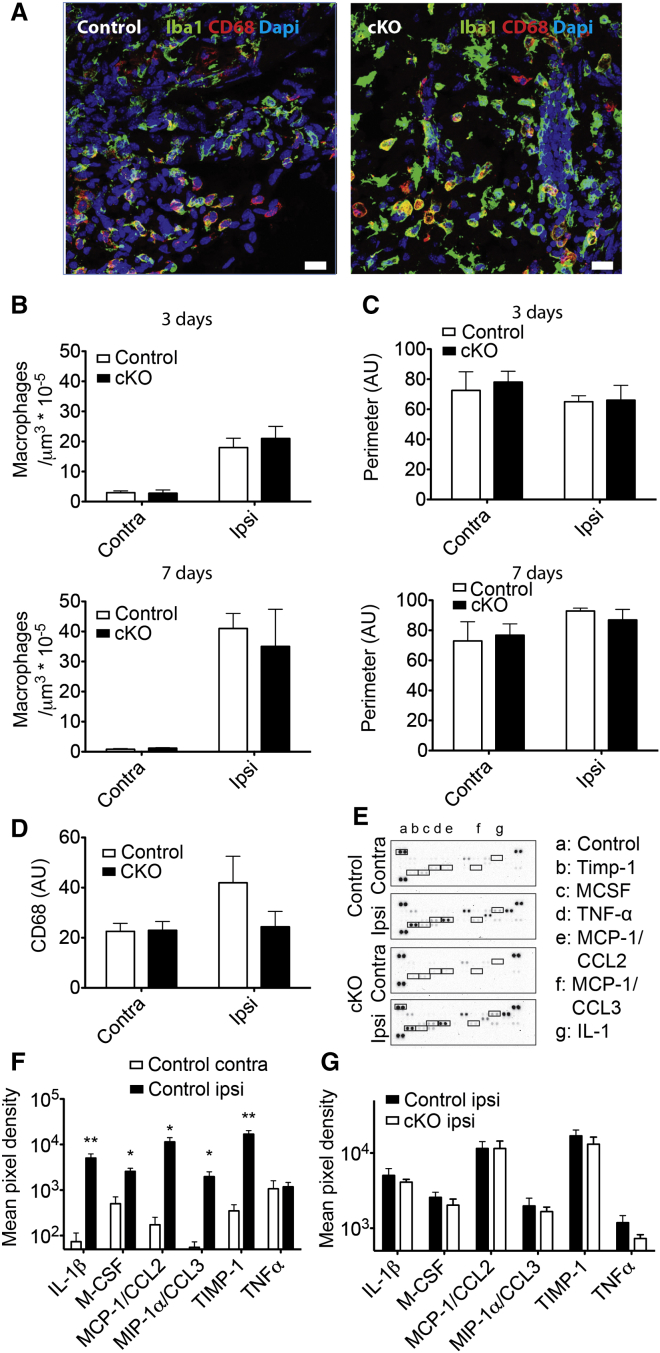
Histological and Biochemical Analysis of Macrophages Recruited at the Nerve Injury (A) Iba1 (red), CD68 (green), and DAPI (blue) immunofluorescence in the ipsilateral nerve cryosections from control and cKO mice, 7 days after injury. Scale bar, 20 μm. (B) Density of Iba1 positive macrophages at the distal side of the nerve injury at 3 and 7 days after injury. n = 3, p < 0.05. (C) Size of Iba1 positive macrophages on the distal side of the nerve injury at 3 and 7 days after injury; n = 3; p < 0.05. (D) CD68 intensity in Iba1 positive macrophages, 7 days after PNL; n = 3; p > 0.05. (E) Cytokine/chemokine array blots incubated with contralateral and ipsilateral sciatic nerve extracts (130 μg) from control and cKO mice, 4 days after injury. (F) Pixel densities of selected cytokine spots in the contralateral and ipsilateral sciatic nerve lysates from control mice. p < 0.05, p < 0.01. n = 3 sets of pooled samples from four mice each. (G) Pixel densities of selected cytokine spots in the ipsilateral sciatic nerve lysates from control and cKO mice 4 days after injury, p > 0.05. Values are mean ± SEM.

To determine whether genetic ablation of TMEM16F perturbed the function of macrophages in the PNL model, we assessed macrophage recruitment, morphology, and activation at the nerve injury site (Figure 3A). In contrast to data from microglia, we observed no significant difference in the number (Figure 3B), size (Figure 3C), or CD68 immunoreactivity (Figure 3D) of macrophages in cKO mice compared to controls. Moreover, release of pro-inflammatory cytokines and chemokines upon nerve injury was also not significantly changed in *LysM*^*Cre*^*::TMEM16F*^*fl/fl*^ mice (Figures 3E–3G). Thus, TMEM16F may play a more prominent role in microglia compared to peripheral macrophages in the PNL model of neuropathic pain.

### TMEM16F Influences Microglia Motility and Engulfment of Neuronal Material

We developed an ex vivo preparation for live imaging of microglia, using a triple transgenic *CX3CR1*^*GFP*^*:: LysM*^*Cre*^*::TMEM16F*^*fl/fl*^, in which GFP is robustly expressed in microglia.

**Figure 4:**
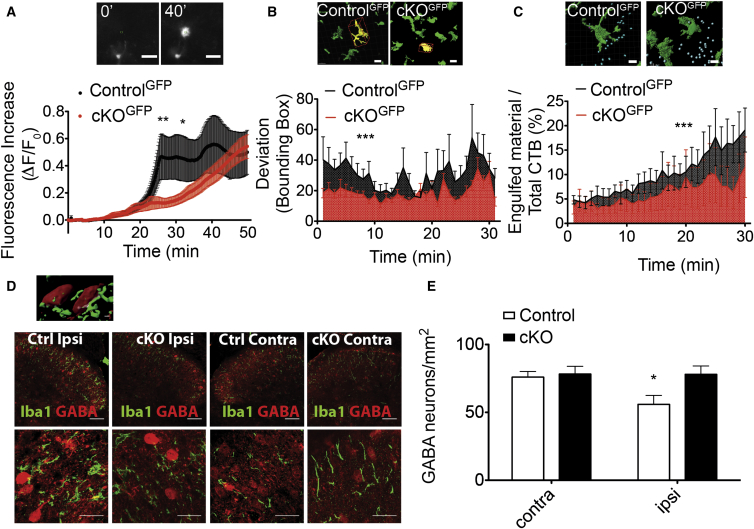
TMEM16F Regulates Microglia Branch Motility and Engulfment of Neuronal Material (A) Top: fluorescence changes in a Control^GFP^ hippocampal slice near the tip (green squares). Bottom: relative fluorescence increase measured in a 10-μm radius from the ATP-containing (3 mM) pipette in acute hippocampal slices from mice of indicated genotypes, n = 9/24 fields in three of seven mice; p < 0.05 from 25 to 34 min. (B) Top: ipsilateral dorsal horn sections from mice of indicated genotypes. The selected segmented GFP positive microglia (yellow) is surrounded by a bounding box (red), which was used for branch motility analysis. Bottom: SD of the bounding box from its mean value over time to quantify microglia branch motility in spinal cord slices, from mice of indicated genotypes.; n = 44/45 cells; p < 0.0001. (C) Segmentation of GFP positive microglia (green) and Alexa-647-labeled neurons, external to microglia (blue) and internalized (pink). Ratio of internalized over total labeled neuronal material to quantify engulfed neuronal terminals by microglia over time. (D) Top: image segmentation: GABA positive neuron (red) colocalizes (white) within Iba1 positive microglia (green) after injury. Bottom: Iba1 (green) and GABA (red) immunofluorescence in the ipsilateral and contralateral dorsal horns from control and cKO mice, 3 days after injury, with zoomed images from lamina I–III in the insets. (E) Density of GABA-positive neurons in the lamina I–III of the dorsal horn of control and cKO mice, 3 days after injury. p < 0.05, n = 5. Values are mean ± SEM. Scale bar, 50 μm (B and C) and 60 μm (D). See Movies S1, S2, S3, and S4.

We examined microglial branch extension in hippocampal slices by applying an ATP puff through a glass micropipette to mimic ATP release from damaged neurons (Davalos et al., 2005). Strikingly, the directed movement of microglia branches was significantly reduced in hippocampal slices from cKO^GFP^ compared to Control^GFP^ mice (Figure 4A; Movie S1). Using tracking analysis of single microglial branches, we observed that the mean elongation velocity of single tracks after an ATP puff was significantly reduced in cKO^GFP^ (1.11 ± 0.03 μm/min) compared to Control^GFP^ mice (1.28 ± 0.06 μm/min; p = 0.017, t test).

As an additional marker of peripheral sensory input into the spinal cord, Cholera Toxin B (CTB)-Alexa 647 was injected intraneurally into the sciatic nerve when performing PNL surgery. During live imaging from spinal cord slices, microglia from cKO^GFP^ mice displayed significantly reduced motility (Figure 4B; Movie S2). Strikingly, we observed that the engulfment of CTB-Alexa-647-labeled nerve terminals was reduced in cKO^GFP^ compared to Control^GFP^ mice (Figure 4C; Movie S3).

In agreement with observations from ex vivo spinal cord preparations, isolated microglia from *Rosa26*^*Cl−sensor*^*::LysM*^*Cre*^*::TMEM16F*^*fl/fl*^ (cKO^Cl−sensor^) mice displayed reduced phagocytosis of fluorescently labeled yeast, compared to control *Rosa26*^*Cl−sensor*^*:: LysM*^*Cre*^*::TMEM16F*^*fl/+*^ (1.6 ± 0.2, n = 96 for control versus 0.9 ± 0.1, n = 87 for cKO, p < 0.05, t test, at 30 min) (Movie S4).

What is the link between impaired microglial phagocytosis and reduced neuropathic pain in TMEM16F cKO mice? Intriguingly, sciatic nerve injury has previously been associated with a selective loss of GABA in the ipsilateral dorsal horn, which, in turn, reduces inhibitory control in the spinal cord and promotes mechanical hypersensitivity (Scholz et al., 2005, Sugimoto et al., 1990). We therefore asked whether loss of GABA might be spared in TMEM16F cKO mice, perhaps as a consequence of the diminished phagocytic capacity of microglia. Utilizing immunohistochemistry for GABA in lumbar spinal cord sections, we observed a significant reduction in GABA staining in the ipsilateral dorsal horn of control mice upon injury (Figure 4D). Strikingly, in TMEM16F knockout mice this reduction was not apparent, and ipsilateral and contralateral sides displayed similar numbers of GABA positive neuron (Figure 4E).

## Discussion

In this study, we explored the significance of high expression levels of TMEM16F in microglia via conditional ablation of the TMEM16F gene in cells of the myeloid lineage. We demonstrate that TMEM16F conditional knockout mice do not develop mechanical hypersensitivity after peripheral nerve injury and that this is associated with a reduced phagocytosis by spinal microglia. Moreover, in both in vivo and ex vivo preparations, microglia exhibit deficits in function that is reflected in their altered morphology, expression of activation markers, and diminished branch motility and phagocytic capacity. Together our data suggest that that the phagocytic activity of microglia is an important component in the cascade of events that lead to altered nociceptive processing in neuropathic pain.

We opted for a conditional genetic strategy to delete TMEM16F and after assessing two Cre driver lines (Clausen et al., 1999, Yona et al., 2013) for reporter gene expression, selected *LysM*^*Cre*^ as this drove expression specifically in microglia in the spinal cord. However, Cre-mediated recombination was not evident in all microglia. We addressed this issue in in vitro studies by using triple transgenic mice expressing Cre-dependent reporter and only selecting fluorescent cells for analysis. In in vivo and ex vivo studies, however, the incomplete deletion of TMEM16F from all microglia will presumably lead to an underestimation of phenotypes. A further complication arising from use of the *LysM*^*Cre*^ driver lines is that recombination will also occur in macrophages. We observed no differences in macrophage number, morphology, or activation at the injury site in the sciatic nerve in cKO versus control mice, or in the expression of inflammatory mediators in the injured nerve. Moreover, in the CFA inflammatory pain model, which does not provoke microglial activation (Li et al., 2013, Lin et al., 2007), nociceptive thresholds were similar between genotypes. These data imply that TMEM16F has a more prominent role in microglia than macrophages in neuropathic pain models. The further development of Cre-driver lines, which selectively target microglia with high efficiency, for example, inducible *Cx3cr1*^*CreER*^ mice (Parkhurst et al., 2013), will allow for a more direct assessment of these issues.

Upon peripheral nerve injury, microglia migrate to the spinal cord, proliferate, and assume an activated state (Gehrmann et al., 1991, Guan et al., 2016). This is accompanied by an increase in P2X4 receptor expression, release of BDNF, and disinhibition of lamina I neurons (Coull et al., 2003, Coull et al., 2005). We therefore asked whether deletion of TMEM16F in microglia would impact upon any of these mechanisms. We observed no decrease in microglial recruitment to the spinal cord (indeed this was significantly increased in TMEM16F cKO mice), and upregulation of P2X4 receptor immunoreactivity in the ipsilateral dorsal horn of TMEM16F cKO mice at levels similar to that seen in control mice.

Other factors have also been demonstrated to contribute to a loss of inhibition in the spinal cord, including a decrease in GABA production and release, and degeneration of GABAergic interneurons (Bráz et al., 2012, Moore et al., 2002, Scholz et al., 2005, Sugimoto et al., 1990). Intriguingly, we observed a reduction in GABA immunoreactivity in lamina I–III of the ipsilateral dorsal horn of control mice, which was absent in the cKO mice. Together with our data on the impaired phagocytic capacity of TMEM16F cKO microglia, we speculate that diminished phagocytosis may spare GABA loss and thus reduce disinhibition and the development of mechanical hypersensitivity. This mechanism could occur independently of complete loss of inhibitory neurons by necrosis or apoptosis and instead happen via selective pruning of GABAergic terminals. Indeed, recent work by Petitjean et al. (2015) has demonstrated that parvalbumin-positive interneurons do not die after peripheral nerve injury but exhibit reduced connectivity with PKCγ neurons in the dorsal horn. Thus, in addition to the well-established role of microglia in releasing bioactive factors (Clark et al., 2007, Clark et al., 2013), their classical phagocytosis function may also contribute to the pathogenesis of neuropathic pain. Further investigation of the integrity of spinal pain circuitry in TMEM16F cKO mice, as well as its role in other cell types (Jiang et al., 2016, Sorge et al., 2015), will shed more light on this issue.

TMEM16F functions as both a calcium dependent ion channel and a phospholipid scramblase. Therefore, an important question that arises from our data is whether deficits in microglial phagocytosis are caused by alterations in ion transport or in phosphatidylserine exposure in cKO cells. Intriguingly, both processes have previously been shown to be important for phagocytosis and ramification in macrophages and microglia suggesting that TMEM16F could utilize multiple mechanisms to modulate microglial function (Callahan et al., 2000, Eder et al., 1998). Recently, TMEM16F was also demonstrated to act downstream of P2X7 receptors and influence immune defense by macrophages (Ousingsawat et al., 2015). While we observed no deficits in macrophage function upon nerve injury in TMEM16F knockout mice, other purinergic receptors such as P2Y12 receptors have been implicated in microglial phagocytosis. Indeed, P2Y12 is expressed exclusively by microglia (Hickman et al., 2013, Kobayashi et al., 2011) and has been demonstrated to play a key role in ATP-mediated branch rearrangement and phagocytosis of injured axons (Haynes et al., 2006, Maeda et al., 2010, Ohsawa et al., 2010). Investigation of interactions between TMEM16F and P2Y12 could therefore be a useful starting point for developing therapeutic strategies that target microglia in neuropathic pain states.

## Experimental Procedures

Details are further described in Supplemental Experimental Procedures.

### Animals

Mice were bred and maintained at the EMBL Mouse Biology Unit, Monterotondo, in accordance with Italian legislation under license from the Italian Ministry of Health. The TMEM16F targeting strategy was designed to allow Cre-mediated excision of the exons 13 of the TMEM16F, resulting in a frameshift mutation in exon 14. The *LysM*^*Cre*^ line (Clausen et al., 1999) was used as a driver line for conditional ablation of TMEM16F. Crosses with *Rosa26*^*tdRFP*^, *Rosa26*^*Cl−sensor*^, and *CX3CR1*^*GFP*^ mouse lines yielded reporters for visual and functional tracking.

### Pain Models and Behavioral Assays

Partial nerve ligation of the left sciatic nerve was performed on mice of both sexes to induce neuropathic pain behavior as described previously (Seltzer et al., 1990). Mice were tested blindly for mechanical allodynia using calibrated von Frey filaments of increasing force applied to the hindpaw and fitting the paw withdrawal probability. Inflammatory pain was induced by intraplantar injection of Complete Freund’s Adjuvant (CFA). To trace central sensory endings, 2-μl injections of 0.5% cholera Toxin-B (CTB) Alexa Fluor 647 conjugate were performed into the sciatic nerve.

### Double RNA Fluorescent In Situ Hybridization and Immunofluorescence

In situ hybridization (ISH) was performed on spinal cord cryosections using a fluorescein-labeled probe generated from a full-length TMEM16F cDNA. Briefly, sections were fixed in 4% paraformaldehyde (PFA), digested with proteinase K for 5 min, acetylated, and hybridized with the probes in 50% formamide, 5 × saline sodium citrate (SSC), 5 × Denhardt’s solution, 500 μg/ml salmon sperm DNA, and 250 μg/ml tRNA overnight at 56°C. After stringent post-hybridization washes, sections were blocked and incubated with mouse anti-fluorescein (Roche; at 1:100) and rabbit anti-Iba1 (Wako; at 1:200), followed by anti-mouse Alexa 555 and anti-rabbit Alexa 488.

### Real-Time PCR

Homogenates of DRG, brain, kidney, L4–L6 segment of the lumbar spinal cord, and lysates of microglia cells were subjected to total RNA extraction and qPCR according to standard protocol. Each mRNA expression level was normalized to ubiquitin or GADPH.

### Western Blotting

Harvested tissues were homogenized on ice in lysis buffer proteinase inhibitor. 10-μg lysates were loaded for gel electrophoresis. Western blot was performed using standard techniques with rabbit anti-TMEM16F (HPA038958, Sigma).

### Immunohistochemistry

Immunohistochemistry was performed on paraformaldehyde-fixed cryosections and free floating sections according to standard protocols. The following primary antibodies were incubated overnight at 4°C: rabbit anti-Iba1 (019-19741, Wako; 2.5 μg/ml), rat anti-CD68 (Abd Biotech; 10 μg/ml), mouse anti-NeuN (1:250), rabbit anti-P2X4 receptor (ab82329, Abcam; 1:200), rabbit anti-RFP (600-401-379, Rockland; 5 μg/ml), and rabbit anti-GABA (A2052, Sigma;1:2,000). For co-staining with RFP or P2X4R or GABA, a mouse goat anti-Iba1 (Novus; 5 μg/ml) was used. Anti-rabbit-, anti-rat, and anti-mouse Alexa 488, 546, or 647 secondary antibodies (2 μg/ml) (Life Technologies) were used. *Z* optical series covered 42 μm of thickness for free floating sections and 12 μm for cryosections with 0.5 μm step.

### Time-Lapse Microscopy of Microglia

Time-lapse imaging of microglia in the culture and spinal cord slices was carried out on a spinning-disk confocal ultraview Vox (Cellular imaging, PerkinElmer) at 37°C and 5% CO_2_. For spinal cord, ex vivo time-lapse *Z* optical series covered 40 μm of thickness with 0.5 μm step. For phagocytosis assays, primary microglia cells expressing endogenous Cl^−^Sensor (Batti et al., 2013) were co-incubated with fluorescent heat-killed *S. cerevisiae* yeast. Images were taken every minute for 30 min.

Time-lapse imaging of microglial branch extension in hippocampal slice from *CX3CR1*^*GFP*^ mice was performed at room temperature during 2–7 hr after cutting. Adenosine 5′-triphosphate magnesium salt (ATP, 2 mM; Sigma-Aldrich) was pressure applied (100 ms; 5 psi) from the glass pipette placed in the stratum radiatum. GFP fluorescence was measured every 10 s for 50 min in a 20-μm diameter area around the pipette tip to quantify the speed of GFP-expressing microglial processes extension.

### Image Analysis

Three-dimensional reconstructions of confocal stacks and surface rendering were performed with Imaris Bitplane Software (Bitplane). Surface, Filament, and Spots Imaris modules were used for segmentation and tracking of microglial cells, microglia processes, and neuron terminals, respectively. Tracking analysis of single microglial processes in the hippocampal slice was performed using ImageJ software.

### Cytokine Arrays

Protein extracts from sciatic nerve fragments were processed with pre-spotted cytokine/chemokine array according to manufacturer’s instructions (R&D Systems, mouse cytokine array panel A, no. ARY006). Signal intensity was analyzed in ImageJ, background subtracted, and averaged on duplicates and between three sets of four mice.

### Statistical Analysis

Statistical significance was determined as p < 0.05 by one-way ANOVA (Figures 2A, 3D, 3F, 3G, and 4C) or two-way ANOVA test (Figures 1A, 2B–2D, 3B, 3C, and 4B) followed by Bonferroni post hoc test, Student’s t test (Figure 4A), χ^2^ test, or Mann Whitney test (Figures 2F and 2G).

## Author Contributions

L.B. and M.S. contributed equally to this work. L.B., M.S., E.M., F.P., and P.A.H. designed experiments. E.M., F.P., and M.S. performed functional in vitro and ex vivo studies. L.B. performed imaging and segmentation analysis. L.B., S.P., and M.S. performed behavioral tests and surgery. L.B., S.P., and M.S. performed biochemistry and immunohistochemistry. F.D.C.R. performed southern blot and RT-PCR. H.W. generated the mouse. E.P. performed tracking analysis. L.B., M.S., P.A.H., F.P., S.D.A., and D.R. supervised the project, L.B., M.S., and P.A.H. co-wrote the manuscript.

## Supplemental Information

Document S1. Supplemental Results, Supplemental Experimental Procedures, and Figures S1–S3

Movie S1. ATP-Induced Branch Extension Is Reduced in cKO Microglia, Related to Figure 4A Representative time-lapse movies showing fluorescent microglia branch motility toward an ATP pipette puff. On the left: CX3CR1GFP:: TMEM16ffl/fl (ControlGFP) On the right: X3CR1GFP::LysMCre::TMEM16ffl/fl (cKOGFP) mice. T= 0 corresponds to start of acquisition; t=4 min corresponds to Mg-ATP application, 5 psi, 100 ms. Green squares indicate the point of the pipette tip.

Movie S2. Reduced Branch Motility of Spinal cKO, Related to Figure 4B Representative Z-stack time lapse movie showing segmented spinal microglia (green and yellow) from a ControlGFPmouse followed by a cKOGFP mouse, and analysis of processes motility using a microglia tracking algorithm over time. One frame every minute for a total of 30 minutes. Scale bar 50 μm.

Movie S3. Reduced Engulfment of Labeled Nerve Terminals in cKO Microglia, Related to Figure 4C 3D view of segmented GFP positive microglia (green) and segmented CTB-Alexa-647-labeled neuronal terminals (cyan) in the dorsal horn of an ipsilateral spinal cord slice 3 days after surgery. Segmented neuronal terminals co-localized with GFP signal are considered internalized (purple).

Movie S4. Reduced Phagocytosis of Fluorescently Labelled Yeast by cKO Microglia, Related to Figure 4 Representative movie of microglia (blue) from LysMCre::TMEM16Ffl/+::Rosa26Cl-Sensor (ControlCl-sensor) (left) and LysMCre::TMEM16Ffl/fl::Rosa26Cl-Sensor (cKOCl-Sensor) (right) mice, incubated with fluorescent yeast (orange) for a phagocytosis assay. One frame every minute for a total of 30 minutes.

Document S2. Article plus Supplemental Information
