# Supplementary material for: TMEM16F Regulates Spinal Microglial Function in Neuropathic Pain States
Source: Cell Rep. 2016 Jun 21;15(12):2608–15. doi: 10.1016/j.celrep.2016.05.039 (PMC4921873; doi:10.1016/j.celrep.2016.05.039)
Supplement: Document S1. Supplemental Results, Supplemental Experimental Procedures, and Figures S1–S3 [file mmc1.pdf]

Cell Reports, Volume *15*

Supplemental Information

TMEM16F Regulates Spinal Microglial Function in Neuropathic Pain States

Laura Batti, Mayya Sundukova, Emanuele Murana, Sofia Pimpinella, Fernanda De Castro Reis, Francesca Pagani, Hong Wang, Eloisa Pellegrino, Emerald Perlas, Silvia Di Angelantonio, Davide Ragozzino, and Paul A. Heppenstall

TMEM16F regulates spinal microglial function in neuropathic pain states

Laura Batti[#], Mayya Sundukova[#], Emanuele Murana, Sofia Pimpinella, Fernanda De Castro Reis, Francesca Pagani, Hong Wang, Eloisa Pellegrino, Emerald Perlas, Silvia Di Angelantonio, Davide Ragozzino, Paul A. Heppenstall

[#]Co-first author

Supplemental Results, related to Fig.1

We designed a targeting construct whereby exon 13 of the TMEM16F gene was flanked by *loxp* sites, allowing for excision of the exon by Cre-mediated recombination, and a subsequent frame-shift mutation in exon 14 (mimicking a human mutation observed in Scott syndrome patients (Suzuki et al., 2010). A *frt* flanked neomycin cassette was introduced to enable selection of correctly targeted ES cells. Positive ES cell clones were identified using southern hybridization, and chimeric mice were obtained that were crossed with Flp-expressing transgenic mice to remove the neomycin cassette.

We first generated ubiquitous *TMEM16F* knockout mice (*TMEM16F*$^{-/-}$) by crossing mice with the *Deleter*^{Cre} line (Schwenk et al., 1995). Further crosses were undertaken to remove the Cre transgene and validation of TMEM16F ablation was achieved using southern hybridization of genomic DNA and western blotting from membrane fraction of spleen homogenates (**Fig. S1B,C**). From crosses of *TMEM16F*$^{+/-}$ mice we observed a reduction in the number of *TMEM16F*$^{-/-}$ offspring compared to expected Mendelian ratios (**Fig.S1D**). Moreover, *TMEM16F*$^{-/-}$ mice had reduced body weight in

comparison to their +/+ or +/- littermates (median value: -10% weight difference, $n=6$; $p> 0.005$). We thus opted for a conditional approach to target TMEM16F in microglia.

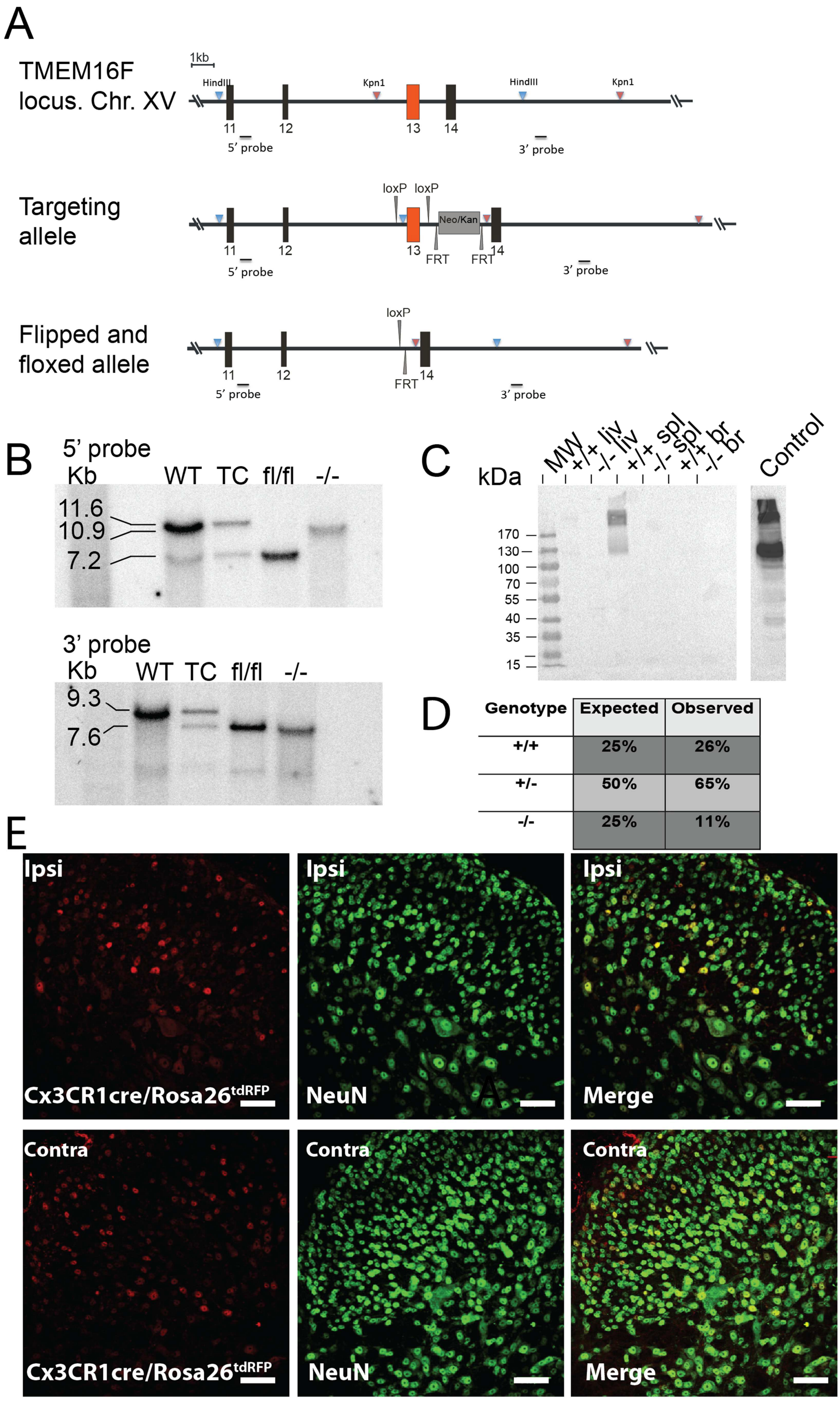
A
TMEM16F locus. Chr. XV
1kb
HindIII
Kpn1
HindIII
Kpn1
11
12
13
14
5' probe
3' probe
Targeting allele
loxP
loxP
Neo/Kan
FRT
FRT
11
12
13
14
5' probe
3' probe
Flipped and floxed allele
loxP
FRT
11
12
14
5' probe
3' probe
B
5' probe
Kb
WT TC fl/fl -/-
11.6
10.9
7.2
3' probe
Kb
WT TC fl/fl -/-
9.3
7.6
C
kDa
MW
+/+ liv
-/- liv
+/+ spl
-/- spl
+/+ br
-/- br
Control
170
130
100
70
55
40
35
15
D
Genotype
Expected
Observed
+/+
25%
26%
+/-
50%
65%
-/-
25%
11%
E
Ipsi
Cx3CR1cre/Rosa26tdRFP
Ipsi
NeuN
Ipsi
Merge
Contra
Cx3CR1cre/Rosa26tdRFP
Contra
NeuN
Contra
Merge

Figure S1, related to Fig. 1

A) Schematic representation of the genomic locus of the TMEM16F gene (top), of the targeted allele (middle) and floxed and flipped alleles (bottom). *Neo* and *Kan*: Neomycin and Kanamycin resistance gene; *loxP* and *FRT* sites are indicated by grey arrowheads; restriction enzymes sites are indicated by blue arrowheads for *HindIII* and red arrowheads for *Kpn1*. B) Southern blot analysis of genomic DNA for indicated genotypes *TC*: targeted cells; fl/fl: floxed mice C) Representative western immunoblot of TMEM16F in three different tissues from indicated genotypes. Control: HEK293 cells transfected with TMEM16F DNA; liv: Liver homogenates; spl: spleen homogenates; br: brain homogenates; D) *TMEM16F* deletion influences the expected Mendelian ratios litters from *TMEM16F*$^{+/-}$ crosses (n=12 litters, $p<0.05$ χ^2 test) E) Representative Z-stack projection of the ipsilateral (top) and contralateral (bottom) dorsal horn of the spinal cord from a *CX3CR1*^{cre}*::Rosa26*^{tdRFP} mouse 5 days after PNL. Anti-RFP (red) , in green anti-NeuN(green) staining. Scale bar: 50 μm.

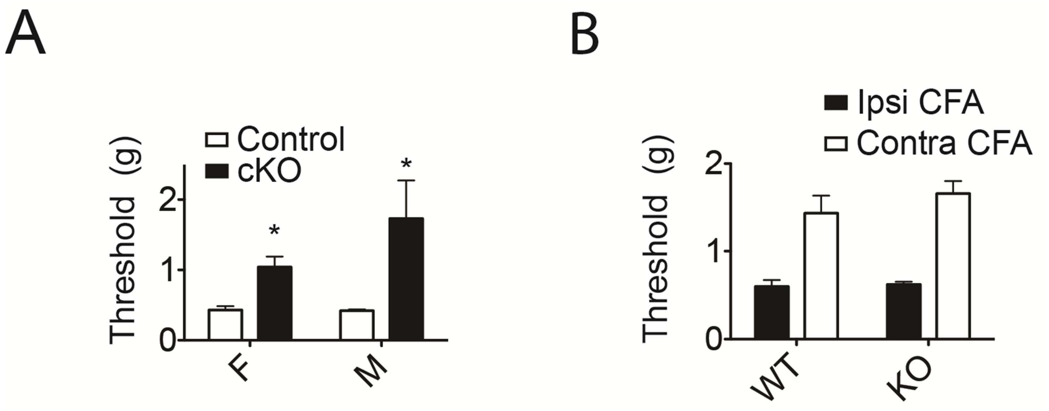

Figure S2, related to Fig. 2

(A) Graph showing the evoked responses to von Frey filaments of the ipsilateral (ipsi) and contralateral (contra) paw in female (F) and male (M) mice seven days after PNL. Data are expressed as paw withdrawal threshold value and are presented as mean ± SEM. N=4/5 for males and 4/3 for females; $p<0.05$ between genotypes, using One-Way ANOVA.

(B) Graph showing the evoked responses to von Frey filaments of the ipsilateral (ipsi) and contralateral (contra) paw two days after Complete Freunds Adjuvant (CFA) injection. Data are expressed as paw withdrawal threshold value and are presented as mean ± SEM. N=6/8; $p>0.05$ between genotypes, using One-Way ANOVA.

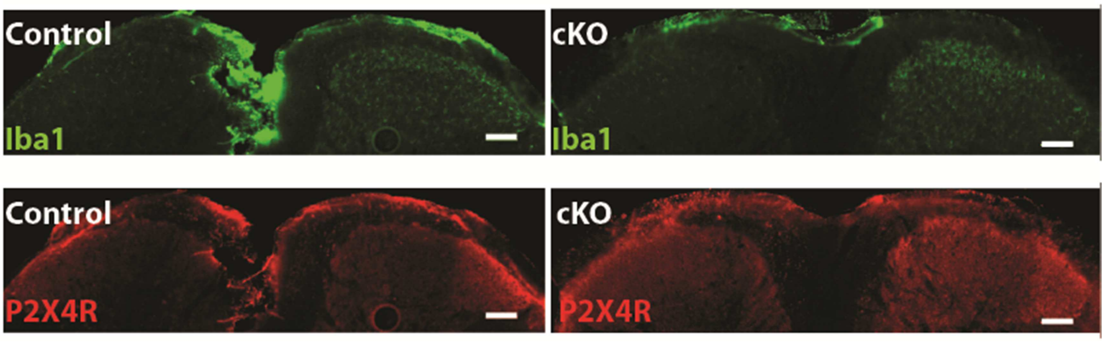

Figure S3, related to Fig. 2

Representative images of lumbar spinal cord slice from TMEM16F$^{fl/fl}$ (control) and *LysM*^{cre}*::TMEM16F*$^{fl/fl}$ (cKO) mice stained with anti-Iba1 (green, top) and anti-P2X4 receptor (red, bottom) antibodies. P2X4 immunoreactivity is increased on the right side of the slice, ipsilateral to PNL, marked by numerous Iba1+ microglia. Scale bar 100 µm. Mean grey levels of P2X4 channel were measured across dorsal horn area (lamina I-III) and normalized to the contralateral side.

Movie S1, related to Fig. 4A

Representative time lapse movies showing fluorescent microglia branch motility towards an ATP pipette puff. *On the left*: *CX3CR1^{GFP}:: TMEM16f$^{fl/fl}$* (Control^{GFP}).*On the right*: *CX3CR1^{GFP}::LysM^{Cre}::TMEM16f$^{fl/fl}$* (cKO^{GFP}) mice.

T= 0 corresponds to start of acquisition; t=4 min corresponds to Mg-ATP application, 5 psi, 100 ms. Green squares indicate the point of the pipette tip.

Movie S2, related to Fig. 4E

Representative Z-stack time lapse movie showing segmented spinal microglia (green and yellow) from a Control^{GFP}mouse followed by a cKO^{GFP} mouse, and analysis of processes motility using a microglia tracking algorithm over time. One frame every minute for a total of 30 minutes. Scale bar 50 μm.

Movie S3 related to Fig. 4G

3D View of segmented GFP positive microglia (green) and segmented CTB-Alexa647 labelled neuronal terminals (cyan) in the dorsal horn of an ipsilateral spinal cord slice 3 days after surgery. Segmented neuronal terminals co-localized with GFP signal are considered internalized (purple).

Movie S4, related to Fig.4

Representative movie of microglia (blue) from *LysM^{Cre}::TMEM16F$^{fl/+}$::Rosa26$^{Cl\text{-}Sensor}$* (Control$^{Cl\text{-}sensor}$) (left) and *LysM^{Cre}::TMEM16F$^{fl/fl}$::Rosa26$^{Cl\text{-}Sensor}$* (cKO$^{Cl\text{-}Sensor}$) (right) mice, incubated with fluorescent yeast (orange) for a phagocytosis assay. One frame every minute for a total of 30 minutes.

Supplemental experimental procedures

Generation of the TMEM16F conditional knockout allele and genotyping

The TMEM16F targeting strategy was designed to allow Cre-mediated excision of the exons 13 of the TMEM16F, resulting in a frame-shift mutation in exon 14. The TMEM16F gene-targeting vector contained a loxP site located 5' of exon 13, a neomycin resistance (neor) gene flanked with two frt sites, and a loxP site located 3' of exon 13.

The targeting construct was transfected into A9 clone ES cells. Southern blotting of individual ES cell clones was used to identify homologous recombinants. DNA was digested with *Kpn1* or *HindIII* and hybridized with a 3` or 5` probe, respectively. Using the 5` probe, three DNA fragments were obtained: a 11600 bp (wild-type) fragment, a 7200 bp (targeted cells and floxed) fragments and 10900 bp (knock out) fragment. Using the 3` probe, two DNA fragments were obtained corresponding to a 9300 bp (wild-type) fragment, 7600 bp (targeted cells and floxed and knock-out) fragments. Positive clones were injected into 8-cell stage embryos to generate mice heterozygous for the targeted allele. Mice were first crossed with FLP-expressing transgenic mice to remove the frt flanked neoR cassette and then to *LysM*^{Cre} or *Deleter*^{Cre} mice. These genetic manipulations resulted in the generation of TMEM16F flipped and floxed alleles, TMEM16F conditional knockout alleles and TMEM16F null alleles, respectively. All analysis was performed on mice that did not carry the FLP or Del^{Cre} alleles.

Mouse genotype was verified by PCR using primers AAACAGTCACACTGGTGGGTGCC (forward for wild-type and LoxP (fl) allele), AGAGCAACTCCGCCAACCCCTTAG(reverse forward for wild-type and LoxP (fl) allele) and TAGACATTCTTTAGTGACAC (forward for null allele) and TGCTATGTTATCAAATACTAGC (reverse for null allele). The presence of the FLP transgene was determined with the following primers;

CCCATTCCATGCGGGGTATCG and GCATCTGGGAGATC ACTGAG. The presence of Cre recombinase was checked with primers GCACTGATTTCGACCAGGTT and GAGTCATCCTTAGCGCCGTA.All primers were purchased from Sigma Aldrich, Italy.

Animals

Mice were bred and maintained at the EMBL Mouse Biology Unit, Monterotondo, in accordance with Italian legislation under license from the Italian Ministry of Health.

TMEM16F conditional KO mice were crossed with the following mouse line: $CX3CR1^{Cre}$(Yona et al., 2013),$LysM^{Cre}$ (Clausen et al., 1999), $Rosa26^{tdRFP}$(Luche et al., 2007), *Cl-sensor* (Batti et al., 2013), $CX3CR1^{GFP}$ (Jung et al., 2000), Avil-$Cre::Rosa26^{SNAPCaaX}$ (Yang et al., 2008).

$LysM^{Cre}::TMEM16F^{fl/fl}$ were referred to as cKO, $TMEM16F^{fl/fl}$ (in the absence of Cre) or $LysM^{Cre}::TMEM16F^{fl/+}$ mice were referred to as controls, $CX3CR1^{GFP}::$ $LysM^{Cre}::TMEM16F^{fl/-}$ and $CX3CR1^{GFP}::LysM^{Cre}::TMEM16F^{fl/fl}$ were referred to as cKO^{GFP}, $CX3CR1GFP::LysM^{Cre}::TMEM16F^{fl/+}$ or $CX3CR1^{GFP}::TMEM16F^{Fl/+}$ (in the absence of *Cre*) mice were referred to as $Control^{GFP}$, $Cre::TMEM16F^{fl/-}::Rosa26^{Cl\text{-}sensor}$ and $Cre::TMEM16F^{fl/fl}::Rosa26^{Cl\text{-}sensor}$ mice were referred to as $cKO^{Cl\text{-}sensor}$, $LysM^{Cre}::TMEM16F^{fl/+}::Rosa26^{Cl\text{-}Sensor}$ and $LysM^{Cre}::TMEM16F^{+/+}::Rosa26^{Cl\text{-}sensor}$ mice were referred to as $Control^{Cl\text{-}Sensor}$.

Pain models and intraneural Injection

Mice of both sexes were subjected to partial nerve ligation of the left sciatic nerve (Seltzer et al., 1990) under isoflurane anaesthesia (Esteve). Briefly, after exposure of the left sciatic nerve at high-thigh level a 7-0 silicon- treated silk suture (Ethicon, Italy) was inserted into the nerve and tightly ligated so that the dorsal 1/3-1/2 of the nerve thickness was trapped in the ligature. In sham operated mice the nerve was left intact and the wound was closed. In all animals, the right leg and sciatic nerve

were untouched; animals were tested three and seven days after surgery. Cholera Toxin-B (CTB) (lifetechnologies) injection was performed right after nerve ligation. The nerve was subjected to serial injection of CTB Alexa Fluor 647 conjugate (0.5%in 2 µl of saline solution).

Intraplantar injection of Complete Freunds Adjuvant (CFA) (Sigma Aldrich, Italy) was used to model inflammatory pain. 20 µl CFA (Heat-killed *Mycobacterium butyricum*,10 mg/ml in mineral oil) was injected into the left hind paw under brief isoflurane anaesthesia. The inflammation was confined to the left paw throughout the observation period. Sham animals were injected with 20 µl of mineral oil.

Behavioral analysis

All mice were habituated to the testing environment for three days. All mice were tested for mechanical hypersensitivity of the hindpaw one day before and three and seven days after surgery. For the inflammatory model animals were tested 48 hours after injection. Mechanical allodynia was measured using the von Frey test. Briefly, von Frey filaments ranging from 0.04 g to 1.2 g of force were applied perpendicularly to the plantar surface of the paw through wire-mesh observation cages. The paw withdrawal threshold (PWT) was determined by sequentially increasing the stimulus strength and fitting percentage withdrawal with sigmoidal function.

Cell culture preparation

Microglial cell cultures were obtained from mixed glia cultures derived from the cerebral cortices of newborn mice (P0-P2). Cortices were digested in 20 U/ml papain (Sigma Aldrich, Italy)for 20 min at 37 °C followed a gentle trituration. The dissociated cells were washed, suspended in DMEM (Lifetchnologies, Italy) supplemented with 10% fetal bovine serum (FBS, Lifetchnologies, Italy), 100 U/ml penicillin and 0.1 mg/ml streptomycin (Lifetchnologies, Italy), and plated (5×10^5cells/cm2) on flasks coated with poly-L-lysine (100 µg/ml) (Sigma Aldrich, Italy). After 7–9 days,

cells were shaken for 2 h at 37 °C to detach and collect microglial cells. These procedures gave almost pure microglial cell populations as previously described (Lauro et al., 2010). Once collected, microglial cells (10^5) were plated on round glass coverslips (12 mm diameter) or on glass bottom dishes (MatTek Corporation, USA) coated with poly-L-lysine (100 µg/ml) and used for experiments after 2-4 days.

Tissue slice preparation

Spinal cord slice were taken from 8-13 weeks old mice. After cervical dislocation a laminectomy was performed to expose the lumbar spinal cord. The L3-L5 segment of spinal cord was freed, embedded on 2% low melting agarose (Promega, USA) in water and incubated in cold HEPES buffer solution of the following composition (in mM): NaCl 140; 193 NaOH 4.55; HEPES 10; KCl 4; Glucose 5; $MgCl_2$ 1; $CaCl_2$ 2. pH 7.4. 120µm transverse free floating sections of L3-L5 spinal cord segment were obtained using Vibratome (Leica VT1000S) and left in HEPES buffer solution to recover for 30 minutes. Slice were then incubated in an imaging chamber and held in place with a U-shaped stainless steel rod. Imaging was carried out excluding the areas damaged by tissue sectioning 30 µm above and beneath the section.

Hippocampal slices were prepared from P30–P40 mice. Animals were decapitated after being anesthetized with halothane, and whole brains were rapidly immersed for 10 min in ice-cold continuously oxygenated (95% O2, 5% CO2; pH 7.4) artificial cerebrospinal fluid (ACSF) containing 125 mM NaCl, 2.5 mM KCl, 1 mM MgCl2, 2 mM CaCl2, 1.125 mM NaH2PO4, 10 mM glucose and 26 mM NaHCO3. Transverse 250-µm hippocampal slices were cut at 4 °C with a vibratome (DSK, Kyoto, Japan) in oxygenated ACSF and allowed to recover for 1 h in oxygenated 10% diluted ACSF (Hypo-ACSF, 270 mOsm). Individual slices were transferred to the recording chamber under the microscope and superfused with oxygenated Hypo-ACSF at the rate of 1.5 ml per min at room temperature (23–25 °C).

Confocal time-lapse microscopy

Time-lapse video microscopy was carried out on a spinning disk confocal ultraviewVox (Cellular imaging, Perkin Elmer) and diode solid lasers (wavelengths: 405, 488, 568 and 647nm) were used as excitation sources. Imaging was performed using a Hamamatsu EMCCD camera (Hamamatsu, Japan). The z-position was controlled by a nano-scan piezo-drive stage (Prior) via the Perkin-Elmer pilot software (VolocityImprovision, UK). Time-lapses were carried out at 37° C and 5%CO_2, unless is specified on the text.

For phagocytosis assays microglia cells expressing Cl-Sensor probe (Batti et al., 2013) were co-incubated with fluorescent heat killed *S. cerevisiae* yeast (2.5 x10^6 cells per dish). Images were taken on a Perkin Elmer spinning disk microscope every minute for 30 minutes.

For spinal cord *ex vivo* time lapse *Z* optical series covered 40 μm of thickness throughout the tissue with 0.5 μm thickness. Images were taken of the dorsal horn of the ipsilateral side of the spinal cord every minute for 30 minutes.

Wide-field time lapse imaging of microglial processes

Wide-field time-lapse fluorescence images in hippocampal slices were acquired at room temperature (24 - 25 °C) using a customized digital imaging microscope. Excitation of GFP was achieved using a 1-nm-bandwidth polychromatic light selector (Till Polychrome V), equipped with a 150 W xenon lamp (Till Photonics, Germany). Fluorescence was visualized using an upright microscope (Axioscope) equipped with a 40x water-immersion objective (AchroplanCarlZeiss, USA) and a digital 12 bit CCD camera system (SensiCam, PCO AG, Germany). All the peripheral hardware control, image acquisition and image processing were achieved using customized software Till Vision v. 4.0 (Till Photonics, Germany). A glass pipette containing adenosine 5'-triphosphate magnesium salt (ATP, 2 mM; Sigma Aldrich) was placed in the stratum radiatum in the center of the recording field. Mg-ATP was pressure applied to the

slices (100 ms; 5 psi) with a Picospritzer III (Parker Instrumentation). Changes in GFP fluorescence distribution was monitored by acquiring a fluorescent stack every 10 seconds for 50 minutes. To quantify the speed of microglial process rearrangement toward the pipette tip, the increase of GFP fluorescence was measured in a circular area centered on the pipette tip (10 µm radius). At each time point the fluorescence increase in the area was calculated as $\Delta F = F\text{-}F_0$, and then divided for F_0 ($\Delta F/F_0$, where F_0 is the average fluorescence before ATP puff) to normalize the difference in basal GFP fluorescence. Slices were used from 2 to 7 hours after cutting. For these experiments *CX3CR1*^{GFP}*:: LysM*^{Cre}*::TMEM16F*$^{fl/-}$ and *CX3CR1*^{GFP}*::LysM*^{Cre}*::TMEM16F*$^{fl/}$*fl* were used as cKO^{GFP} and *CX3CR1*^{GFP}*::TMEM16F*$^{Fl/+}$ and *CX3CR1*^{GFP}*::TMEM16F*$^{Fl/fl}$ (in the absence of Cre) mice were used as Control^{GFP}.

Tracking analysis of single microglial process

All images were processed using ImageJ software. Images stacks were exported as .avi files to enable manual cell processes tracking on the ImageJ “Manual Tracking” plug-in (http://imagej.nih.gov/ij/plugins/track/track.html). To obtain quantitative analysis of tracks parameters, data were analyzed with ImageJ and Origin 7 (OriginLab Co.) software. Stacks were first background subtracted to optimize contrast. To obtain x-y coordinates of single processes, track positions were transferred into a new coordinate system, in which the ATP-containing pipette tip was set as origin (x = 0, y = 0). For each moving process (i), with position vector Ri(t), the change in position from one frame to the next (ΔRi(t)), and the instantaneous velocity (vi(t)) were given by ΔRi(t) = Ri(t+Δt)− Ri(t), and vi(t) = ΔRi(t)/Δt respectively, where Δt is the elapsed time among the two frames. The mean elongation velocity of each process was calculated as <v>=dx/dt, expressed in µm/min, defining dx as the mean accumulated distance of each process i sampled within the time interval dt.

In Situ Hybridization

Cells grown on coverslips were fixed in 4% PFA for 30 min, washed in PBS and permeabilized by overnight incubation in 70% ethanol at 4C. In situ hybridization was carried out using an in vitro transcribed probe generated from the full coding region of TMEM 6F. Cells were acetylated and hybridized with the probe in 50% formamide, 5X SSC, 5x Denhardt's solution, 500 ug/ml salmon sperm DNA, and 250 ug/ml tRNA overnight at 56 C. After post-hybridization washes with 50% formamide, 2X SSC at 52.5 C, and with 2X SSC at ambient temperature, sections were blocked and incubated overnight with anti-digoxigenin-AP (Roche; at 1:1000). Signal detection was done using NBT/BCIP substrate.

RNA fluorescence in situ hybridization and immunofluorescence

Spinal cords were collected, fresh frozen in OCT and sectioned at 20 µm onto Superfrost Plus slides. In situ hybridization was performed using a Fluorescein-labeled probe generated from a full-length TMEM16F cDNA. Briefly, sections were fixed in 4% paraformaldehyde, digested with proteinase K for 5 min, acetylated, and hybridized with the probes in 50% formamide, 5X SSC, 5x Denhardt's solution, 500 ug/ml salmon sperm DNA, and 250 ug/ml tRNA overnight at 56º C. After post-hybridization washes with 50% formamide, 2X SSC at 45 C, and with 2X SSC at ambient temperature, sections were blocked and incubated overnight with mouse anti-fluorescein (Roche; at 1:100) and was detected by a donkey anti-mouse Alexa 555. The sections were then incubated with rabbit anti-Iba1 (Wako; at 1:200), followed by donkey anti-rabbit Alexa 488.

Real Time PCR

Homogenates of DRGs, Brain, Kidney, L4–L6 segment of the lumbar spinal cord and lysates of microglia cells were subjected to total RNA extraction with an RNeasy Micro Kit (Qiagen) according to the manufacturer's instruction. mRNA was reverse

transcribed to cDNA by Superscript II reverse transcription (Life Technology), using Oligo(dT) primers. Quantitative PCR was performed on a LightCycler 480 PCR instrument (Roche) using SYBR green I master (Roche). The primers were as follows: TMEM16A primers (forward: 5'-GAGGCCAGTAGCCATCAGAG-3'), (reverse: 5'-TCTGGAAGTCGCTGACATTG-3'), TMEM16B (forward: 5'-CGGATATCCCCACTGACATC-3'), (reverse: 5'-ACCCTGAGGATGCTATGCTG-3') TMEM16C (forward: 5'-CGAAAGCCAAGTAAGCCAAG-3'), (reverse: 5'-AAGACTGTGGCCCATATTGC-3'), TMEM16D (forward: 5'-CGACTTCATCCCTCGCTTAG-3'), (reverse: 5'-TAGGGTGCCAAAGAGTGAGG-3'), TMEM16E (forward: 5'-CCAGGAGTATGAGAGCAGCC-3'), (reverse: 5'-AATGATGGTTAGCTGGGTGG-3'), TMEM16F (forward: 5'-GCACTTGGAGCAAAGAGGTC-3'), (reverse: 5'-TGCTGTAGCTCAACGGTGTC-3'), TMEM16G (forward: 5'-AAGGTACTTCGGGGAGAAGG-3'), (reverse: 5'-AAGAACAGTCGGAGCACAGC-3'), TMEM16H (forward: 5'-CAAGGTTATGCTAGCCCTGC-3'), (reverse: 5'-TCTTTTAGGCGGTCCATGTC-3'), TMEM16I (forward: 5'-ACCTTGTGGAAATCCGTCTG-3'), (reverse: 5'-ACGGGCCATAGTGGTACTTG-3'), TMEM16J (forward: 5'-ACCTTGTGGAAATCCGTCTG-3'), (reverse: 5'-ACGGGCCATAGTGGTACTTG-3') for RT-PCR of TMEM16 family members. All primers were purchased from Sigma Aldrich, Italy. Each mRNA expression level was normalized to ubiquitin or GADPH.

Western blotting

Harvested tissues were homogenized on ice in buffer containing 10 mM Hepes, 1mM EDTA, 250 mM sucrose and proteinase inhibitor using a Politron homogenizer. Homogenates were centrifuged at 1500 rpm for 5 min at 4 ºC. For the membrane fraction, 100 mM NaCO3 (pH 11.2) was then added to the supernatant and shaken for 45 min at 4 ºC. Lysates were incubated in sample buffer for 30 min at 37 ºC. Lysates from HEK293 cells expressing TMEM16F were used as positive control. 10

μg lysates were loaded for gel electrophoresis. Proteins were transferred to a nitrocellulose membrane, and incubated with rabbit anti-TMEM16F (HPA038958, Sigma) antibody in 0.1% Tween TBS + 5% dry nonfat milk, and HRP-linked donkey anti-rabbit IgG (NA934V, GE Healthcare) secondary antibody. Detection was performed using the ECL Western blot chemiluminescent reagent (RPN2106, GE Healthcare).

Immunohistochemistry

Mice were anesthetized with 2.5% Avertin and subjected to intracardiac perfusion with fresh 4% paraformaldehyde in PBS. Spinal cord and sciatic nerve were dissected and postfixed with 4% paraformaldehyde in PBS for 120 and 30 minutes, respectively. L3-L5 Lumbar segments of the spinal cord were embedded in 2% low melting agarose in water and 50μm transverse free-floating section were obtained using a Vibratome (Leica). Free floating section were permeabilized in 0.5% Triton-X in PBS for 1 hour at room temperature. Unspecific binding was blocked using a 1 hour incubation with 1% BSA in 0.5% Triton X-100. Nerves were cryoprotected in 30% sucrose PBS, and cut on a cryostat (18 μm; Leica). Cryosections were permeabilized using 0.2% Triton X-100 in TBS for 10 minutes and blocked with 5% normal goat serum in 0.1% Tween-20 TBS for 30 minutes at room temperature. The following primary antibodies were incubated overnight at 4°C at the described dilution: rabbit anti-Iba1 (019-19741, Wako; 2.5 μg/ml), rat anti-CD68 (Abd Biotech; 10 μg/ml), mouse anti-NeuN (1:250), rabbit anti-P2X4 receptor (ab82329, Abcam; 1:200), rabbit anti-RFP (600-401-379, Rockland; 5 μg/ml), rabbit anti-GABA (A2052, Sigma;1:2000). For co-staining with RFP or P2X4R, a mouse goat anti-Iba1 (Novus; 5 μg/ml) was used. Anti-rabbit-, anti-rat and anti-mouse Alexa-488, -546 or -647 secondary antibodies (2 μg/ml) (Life Technologies) were applied and incubated for 90 minutes at room temperature. Dapi (5 μg/ml) was applied for 20 minutes and washed by PBS. Sections were mounted on slides using Pro-long Gold (Life Technologies). Confocal microscopy was performed using a TCS-SP5 (Leica) Laser

Scanning System. For free floating slice imaging, *Z* optical series covered 42 μm of thickness throughout the tissue with 0.5 μm thickness. For imaging of cryosections *Z* optical series covered 12 μm of thickness throughout the tissue with 0.5 μm thickness. Low magnification spinal cord tiled images were acquired using wide-field microscope (Leica Microsystems).

Confocal Image analysis

Three-dimensional reconstructions of confocal stacks and surface rendering were generated using Imarisbitplane© Software (Bitplane, Zurich, Switzerland). *Surface*, *Filament* and *Spots*Imaris modules were used for segmentation and tracking of microglial cells, microglia processes and neuron terminals, respectively. For microglia branch motility analysis, the volume of rectangular cuboid containing single objects was measured for each timepoint, using IMARIS *Surface* module. For detection of internalized neuronal terminals by microglia, objects identified by the *spots* module were filtered by mean intensity with a thresholded green (microglia) intensity. Volocity 3D image analysis software (Perkin Elmer) was used for three-dimensional reconstructions and quantitative analysis of confocal stack of nerve sections.

Cytokine arrays

Mice at day 4 after PNL induction were trascardially perfused with PBS, and sciatic nerve fragments were excised and immediately frozen in liquid nitrogen. Samples were thawed, homogenized and protein extracted. Contralateral and ipsilateral sciatic nerve protein extracts were pooled together from 4 mice to produce 130 μg protein. Each sample was incubated with a separate pre-spotted membrane of 40 cytokines/chemokines and processed according to manufacturer’s instructions (R&D Systems, mouse cytokine array panel A, no. ARY006). The signal was revealed with streptavidin-HRP-conjugated secondary antibody and chemiluminescence. Intensity

of selected dots was analyzed in ImageJ, and mean pixel density for each protein was calculated by subtracting background and averaging the duplicates.

Statistical Analysis

Data analysis was performed with PRISM (GraphPad), Origin 6.1 (OriginLab Co.) and SigmaPlot (SyStat). All data are reported as mean ± s.e.m. Statistical significance was determined by one-way ANOVA test followed by Bonferroni *post hoc* test in case of significance, two-way ANOVA, Student's t-test, χ^2 test, Kolmogorov-Smirnov or Mann Whitney test.

Batti, L., Mukhtarov, M., Audero, E., Ivanov, A., Paolicelli, R.C., Zurborg, S., Gross, C., Bregestovski, P., and Heppenstall, P.A. (2013). Transgenic mouse lines for non-invasive ratiometric monitoring of intracellular chloride. Frontiers in molecular neuroscience *6*, 11.

Clausen, B.E., Burkhardt, C., Reith, W., Renkawitz, R., and Forster, I. (1999). Conditional gene targeting in macrophages and granulocytes using LysMcre mice. Transgenic research *8*, 265-277.

Jung, S., Aliberti, J., Graemmel, P., Sunshine, M.J., Kreutzberg, G.W., Sher, A., and Littman, D.R. (2000). Analysis of fractalkine receptor CX(3)CR1 function by targeted deletion and green fluorescent protein reporter gene insertion. Molecular and cellular biology *20*, 4106-4114.

Lauro, C., Cipriani, R., Catalano, M., Trettel, F., Chece, G., Brusadin, V., Antonilli, L., van Rooijen, N., Eusebi, F., Fredholm, B.B.*, et al.* (2010). Adenosine A1 receptors and microglial cells mediate CX3CL1-induced protection of hippocampal neurons against Glu-induced death. Neuropsychopharmacology : official publication of the American College of Neuropsychopharmacology *35*, 1550-1559.

Luche, H., Weber, O., Nageswara Rao, T., Blum, C., and Fehling, H.J. (2007). Faithful activation of an extra-bright red fluorescent protein in "knock-in" Cre-reporter

mice ideally suited for lineage tracing studies. European journal of immunology *37*, 43-53.

Schindelin, J., Arganda-Carreras, I., Frise, E., Kaynig, V., Longair, M., Pietzsch, T., Preibisch, S., Rueden, C., Saalfeld, S., Schmid, B.*, et al.* (2012). Fiji: an open-source platform for biological-image analysis. Nature methods *9*, 676-682.

Schneider, C.A., Rasband, W.S., and Eliceiri, K.W. (2012). NIH Image to ImageJ: 25 years of image analysis. Nature methods *9*, 671-675.

Schwenk, F., Baron, U., and Rajewsky, K. (1995). A cre-transgenic mouse strain for the ubiquitous deletion of loxP-flanked gene segments including deletion in germ cells. Nucleic Acids Res 23, 5080-5081.

Seltzer, Z., Dubner, R., and Shir, Y. (1990). A novel behavioral model of neuropathic pain disorders produced in rats by partial sciatic nerve injury. Pain *43*, 205-218.

Suzuki, J., Umeda, M., Sims, P.J., and Nagata, S. (2010). Calcium-dependent phospholipid scrambling by TMEM16F. Nature *468*, 834-838.

Yang, Y.D., Cho, H., Koo, J.Y., Tak, M.H., Cho, Y., Shim, W.S., Park, S.P., Lee, J., Lee, B., Kim, B.M.*, et al.* (2008). TMEM16A confers receptor-activated calcium-dependent chloride conductance. Nature *455*, 1210-1215.

Yona, S., Kim, K.W., Wolf, Y., Mildner, A., Varol, D., Breker, M., Strauss-Ayali, D., Viukov, S., Guilliams, M., Misharin, A.*, et al.* (2013). Fate mapping reveals origins and dynamics of monocytes and tissue macrophages under homeostasis. Immunity *38*, 79-91.
