# Supplementary figures and images for: TMEM16F Regulates Spinal Microglial Function in Neuropathic Pain States
Source: Cell Rep. 2016 Jun 21;15(12):2608–15. doi: 10.1016/j.celrep.2016.05.039 (PMC4921873; doi:10.1016/j.celrep.2016.05.039)

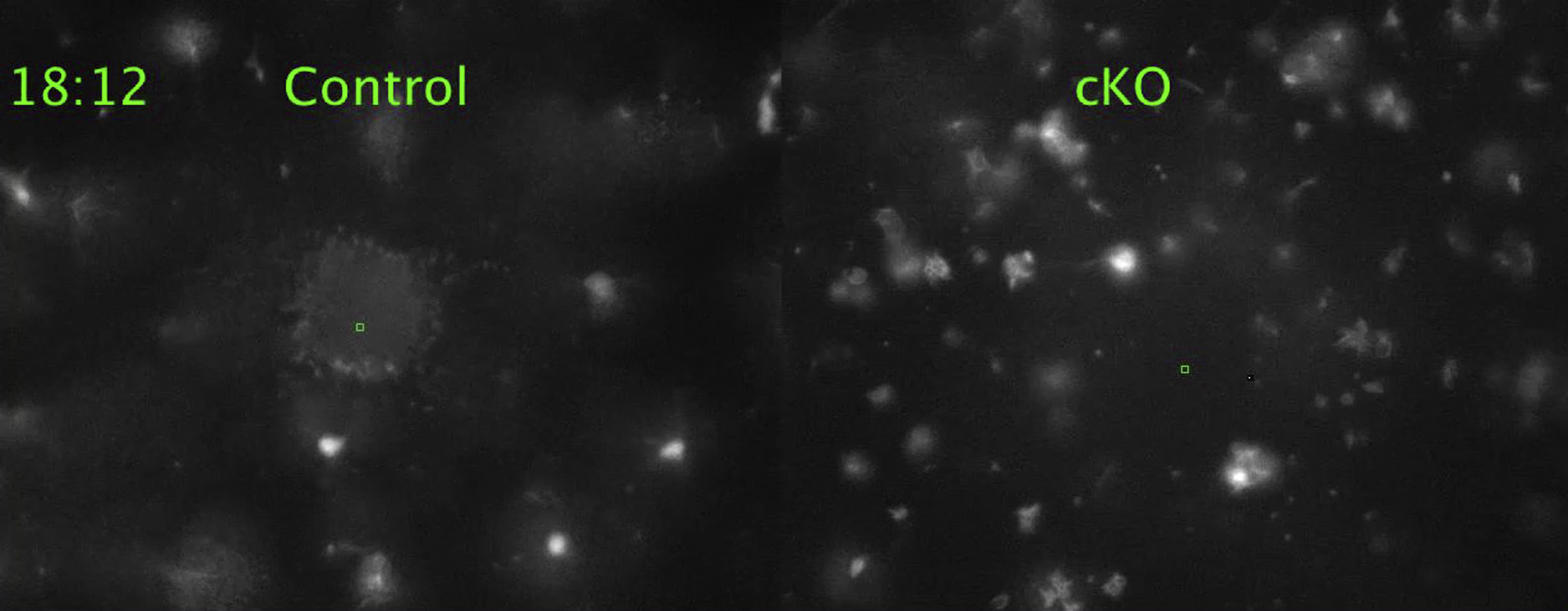

Supplement: Movie S1. ATP-Induced Branch Extension Is Reduced in cKO Microglia, Related to Figure 4A — Representative time-lapse movies showing fluorescent microglia branch motility toward an ATP pipette puff. On the left: CX3CR1GFP:: TMEM16ffl/fl (ControlGFP) On the right: X3CR1GFP::LysMCre::TMEM16ffl/fl (cKOGFP) mice. T= 0 corresponds to start of acquisition; t=4 min corresponds to Mg-ATP application, 5 psi, 100 ms. Green squares indicate the point of the pipette tip. [file mmc2.jpg]

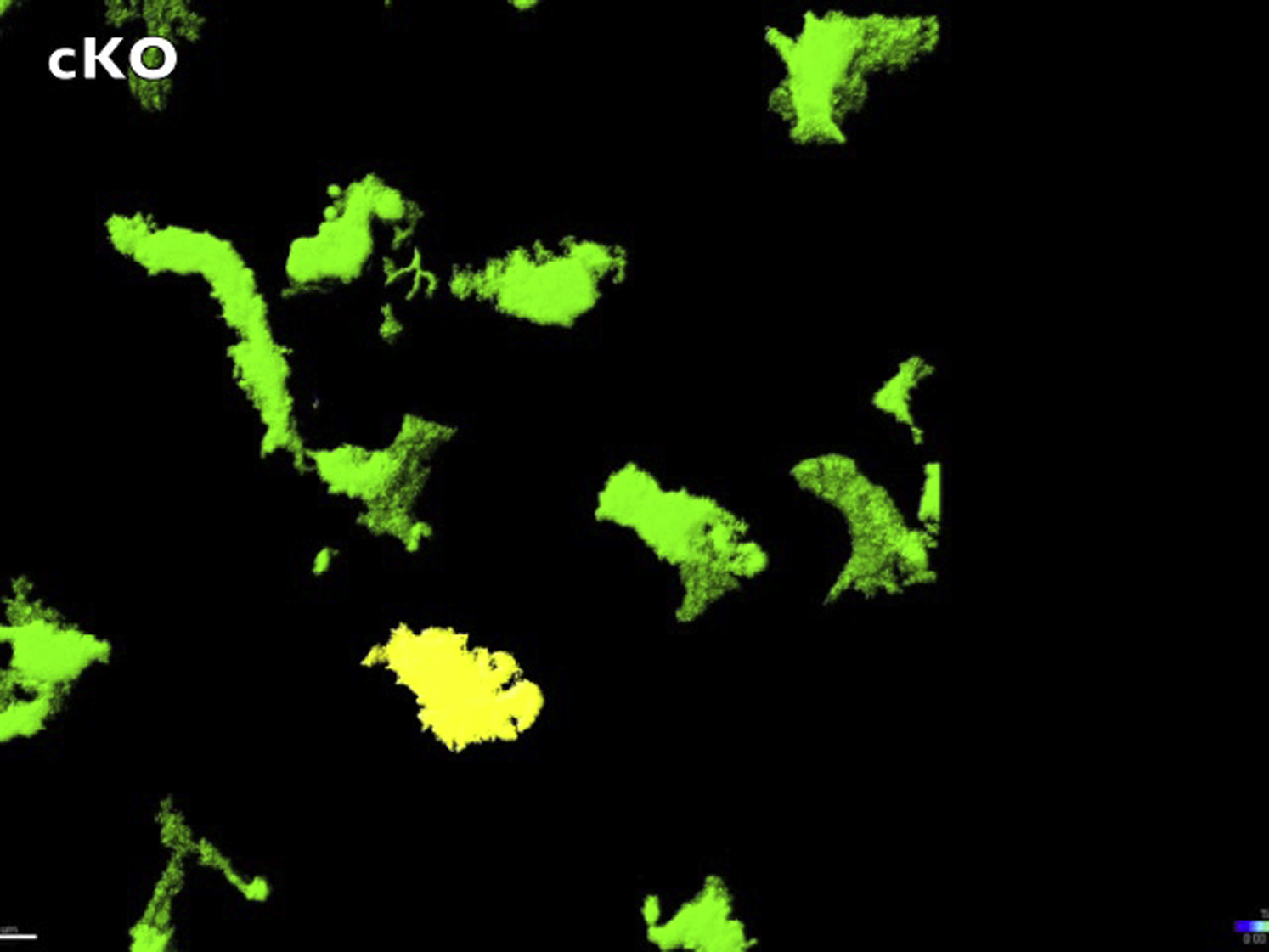

Supplement: Movie S2. Reduced Branch Motility of Spinal cKO, Related to Figure 4B — Representative Z-stack time lapse movie showing segmented spinal microglia (green and yellow) from a ControlGFPmouse followed by a cKOGFP mouse, and analysis of processes motility using a microglia tracking algorithm over time. One frame every minute for a total of 30 minutes. Scale bar 50 μm. [file mmc3.jpg]

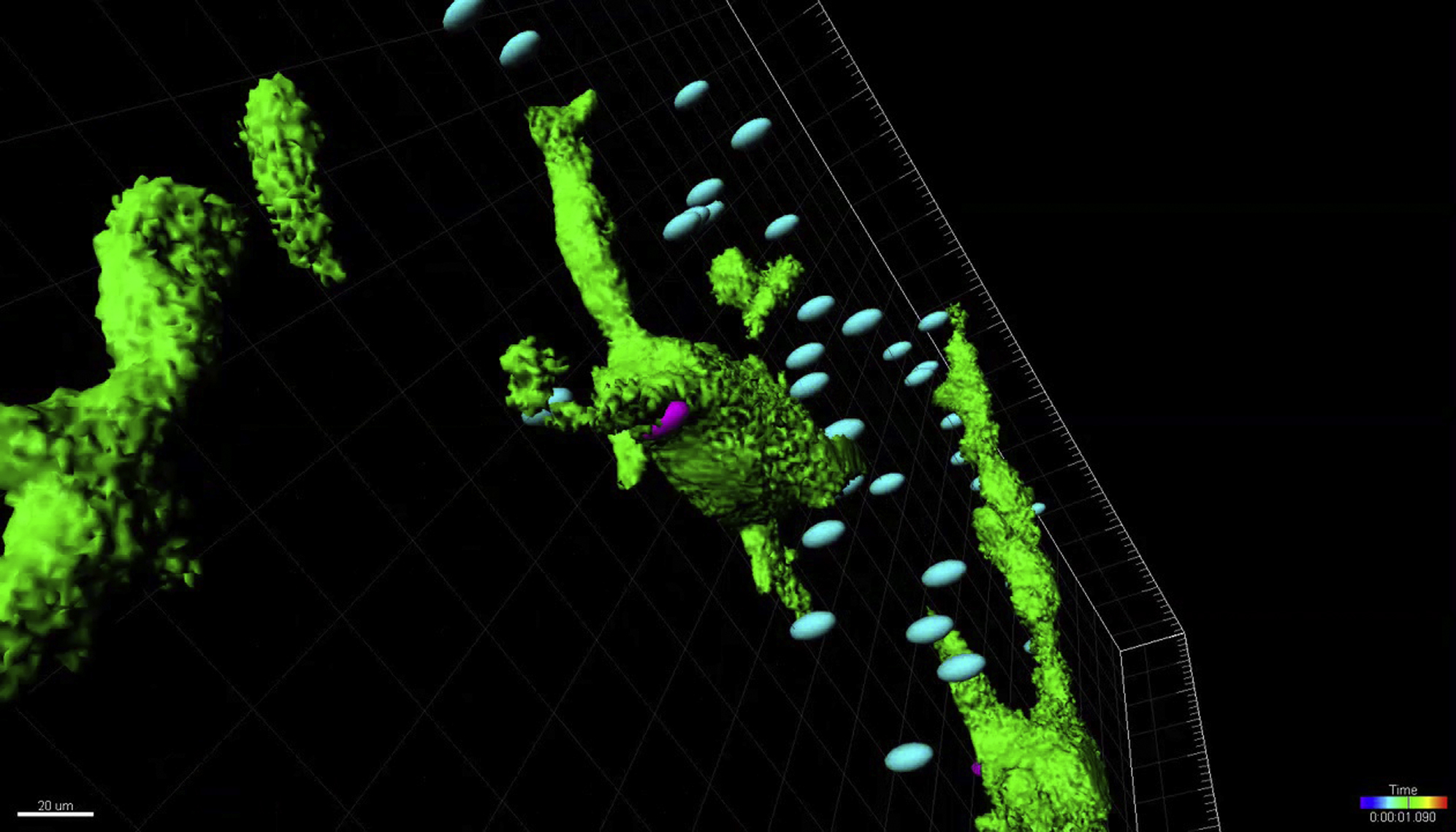

Supplement: Movie S3. Reduced Engulfment of Labeled Nerve Terminals in cKO Microglia, Related to Figure 4C — 3D view of segmented GFP positive microglia (green) and segmented CTB-Alexa-647-labeled neuronal terminals (cyan) in the dorsal horn of an ipsilateral spinal cord slice 3 days after surgery. Segmented neuronal terminals co-localized with GFP signal are considered internalized (purple). [file mmc4.jpg]

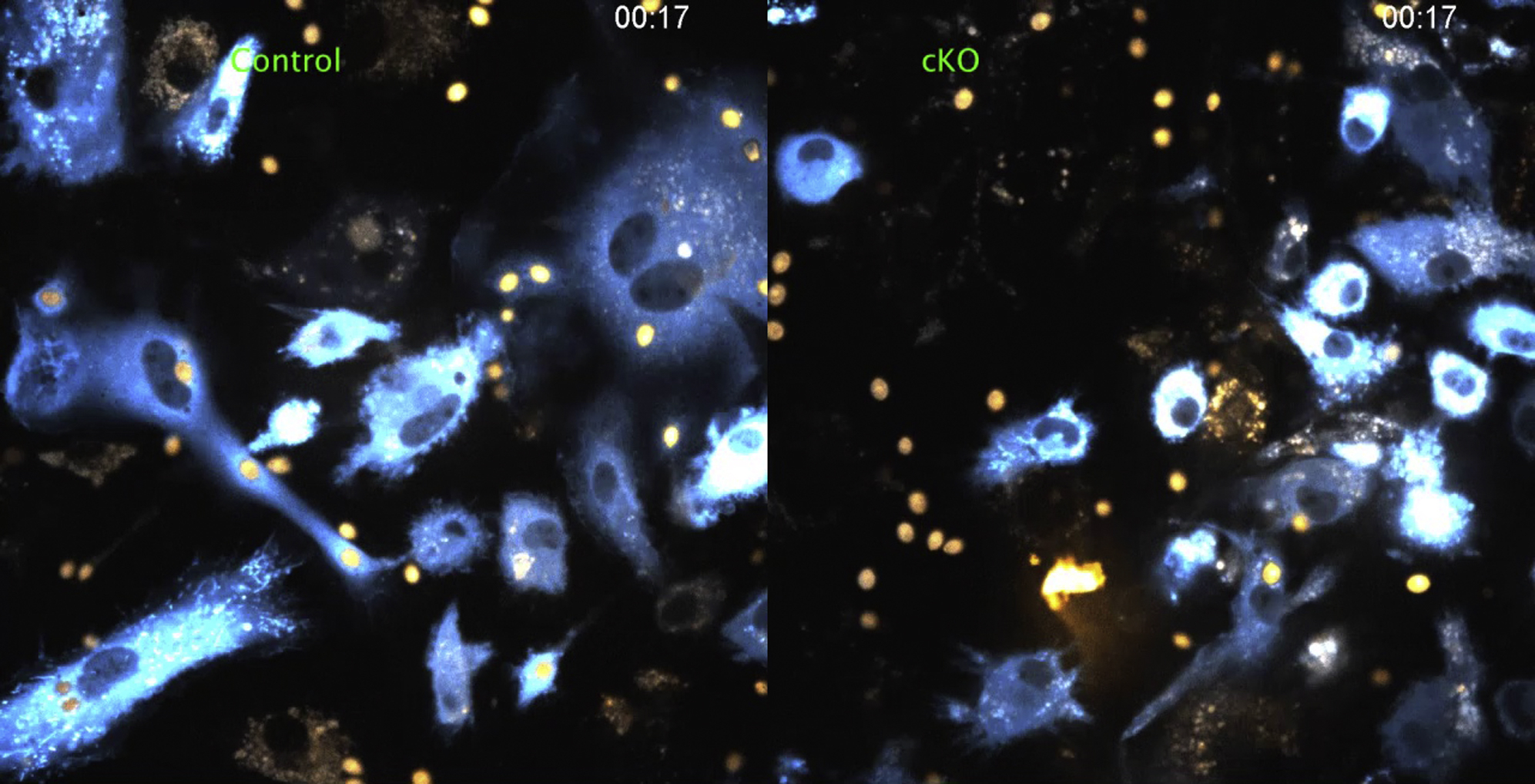

Supplement: Movie S4. Reduced Phagocytosis of Fluorescently Labelled Yeast by cKO Microglia, Related to Figure 4 — Representative movie of microglia (blue) from LysMCre::TMEM16Ffl/+::Rosa26Cl-Sensor (ControlCl-sensor) (left) and LysMCre::TMEM16Ffl/fl::Rosa26Cl-Sensor (cKOCl-Sensor) (right) mice, incubated with fluorescent yeast (orange) for a phagocytosis assay. One frame every minute for a total of 30 minutes. [file mmc5.jpg]
