# Supplementary material for: TMEM16F Regulates Spinal Microglial Function in Neuropathic Pain States
Source: Cell Rep. 2016 Jun 21;15(12):2608–15. doi: 10.1016/j.celrep.2016.05.039 (PMC4921873; doi:10.1016/j.celrep.2016.05.039)
Supplement: Document S2. Article plus Supplemental Information [file mmc6.pdf]

Cell Reports

Report

TMEM16F Regulates Spinal Microglial Function in Neuropathic Pain States

Graphical Abstract

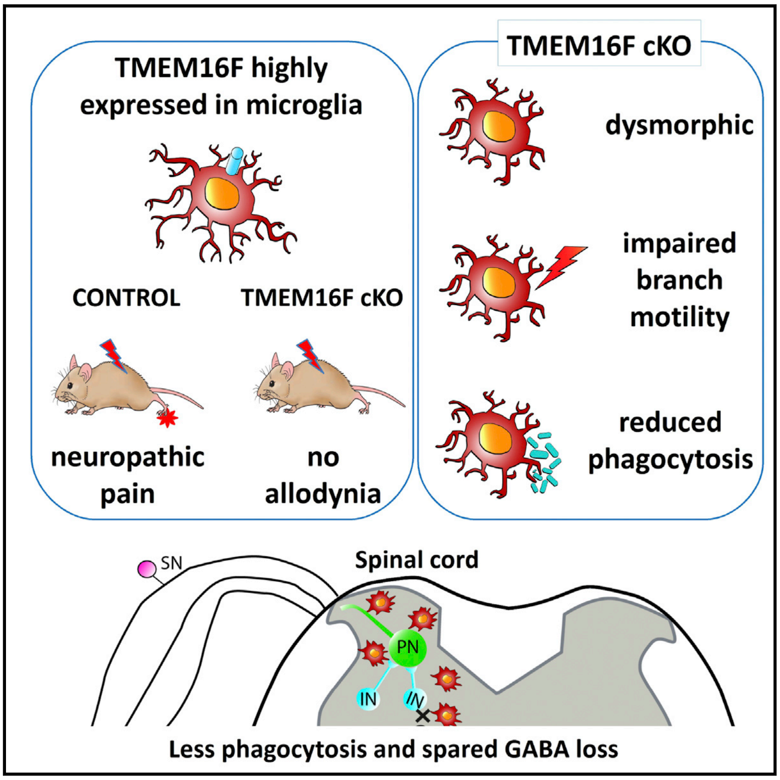

Highlights

- Microglial TMEM16F channels are required for neuropathic pain development in mice
- TMEM16F-deficient microglia display deficits in process motility and phagocytosis
- Deleting TMEM16F spares injury-induced loss of spinal cord GABA immunoreactivity
- Microglial phagocytosis may contribute to neuropathic pain development

Authors

Laura Batti, Mayya Sundukova, Emanuele Murana, ..., Silvia Di Angelantonio, Davide Ragozzino, Paul A. Heppenstall

Correspondence

batti@embl.it (L.B.),
paul.heppenstall@embl.it (P.A.H.)

In Brief

Batti et al. suggest that microglial phagocytosis may contribute to development of neuropathic pain. Mice with conditional TMEM16F ablation in microglia do not develop mechanical pain upon nerve injury. The authors show that TMEM16F-deficient microglia display deficits in process motility and phagocytosis.

Batti et al., 2016, Cell Reports *15*, 2608–2615
June 21, 2016 © 2016 The Authors.
http://dx.doi.org/10.1016/j.celrep.2016.05.039

OPEN ACCESS
CellPress

Cell Reports
Report

TMEM16F Regulates Spinal Microglial Function in Neuropathic Pain States

Laura Batti,[1,7,*] Mayya Sundukova,[1,7] Emanuele Murana,[4] Sofia Pimpinella,[1] Fernanda De Castro Reis,[1] Francesca Pagani,[3] Hong Wang,[5] Eloisa Pellegrino,[4] Emerald Perlas,[1] Silvia Di Angelantonio,[3,4] Davide Ragozzino,[4,6] and Paul A. Heppenstall[1,2,*]
[1]EMBL Mouse Biology Unit, Via Ramarini 32, Monterotondo 00015, Italy
[2]Molecular Medicine Partnership Unit (MMPU), 69117 Heidelberg, Germany
[3]Center for Life Nanoscience, Istituto Italiano di Tecnologia, Viale Regina Elena 291, 00161 Rome, Italy
[4]Istituto Pasteur-Fondazione Cenci Bolognetti and Department of Physiology and Pharmacology, Sapienza University of Rome, Piazzale Aldo Moro, 5 00185 Rome, Italy
[5]Pharmacology Institute, University of Heidelberg, Im Neuenheimer Feld 366, 69120 Heidelberg
[6]IRCCS Neuromed, Via Atinese, Pozzilli 86077, Italy
[7]Co-first author
*Correspondence: batti@embl.it (L.B.), paul.heppenstall@embl.it (P.A.H.)
http://dx.doi.org/10.1016/j.celrep.2016.05.039

SUMMARY

Neuropathic pain is a widespread chronic pain state that results from injury to the nervous system. Spinal microglia play a causative role in the pathogenesis of neuropathic pain through secretion of growth factors and cytokines. Here, we investigated the contribution of TMEM16F, a protein that functions as a Ca^{2+}-dependent ion channel and a phospholipid scramblase, to microglial activity during neuropathic pain. We demonstrate that mice with a conditional ablation of TMEM16F in microglia do not develop mechanical hypersensitivity upon nerve injury. In the absence of TMEM16F, microglia display deficits in process motility and phagocytosis. Moreover, loss of GABA immunoreactivity upon injury is spared in TMEM16F conditional knockout mice. Collectively, these data indicate that TMEM16F is an essential component of the microglial response to injury and suggest the importance of microglial phagocytosis in the pathogenesis of neuropathic pain.

INTRODUCTION

Neuropathic pain is a widespread and debilitating clinical condition that is triggered by a lesion in the nervous system. (Campbell and Meyer, 2006; Costigan et al., 2009). It is becoming increasingly apparent that spinal microglia play a causative role in the pathogenesis of neuropathic pain (Scholz and Woolf, 2007). Peripheral nerve injury is associated with a pronounced recruitment of microglia to the spinal cord, and a conversion of these cells into a reactive state (Gehrmann et al., 1991), whereby they increase synthesis and release of bioactive molecules (Clark et al., 2007, 2013).

The most characterized mechanism through which microglia contribute to neuropathic pain involves a molecular pathway that is dependent upon upregulation of microglial purinergic P2X4 receptors (Beggs et al., 2012; Tsuda et al., 2003) and increased release of brain-derived neurotrophic factor (BDNF) from microglia, which acts on dorsal horn lamina I neurons to shift their transmembrane anion gradient (Coull et al., 2003, 2005). As a result, inhibitory synaptic signaling through $GABA_A$ and glycine receptors is diminished. Furthermore, loss of inhibition is exacerbated by reduced production and release of spinal GABA (Lever et al., 2003; Moore et al., 2002) and injury-induced loss of GABAergic interneurons in the dorsal horn (Scholz et al., 2005; Sugimoto et al., 1990).

Microglia also have a well-established role in the detection and removal of apoptotic neuronal material (Davalos et al., 2005; Fu et al., 2014; Sierra et al., 2013). Upon injury, activated microglia converge on the dorsal horn in response to chemokine and ATP signaling and survey and modify sensory afferent input for damage via probing and extension of their processes (Davalos et al., 2005). Critical to this function is the activation of ion channels, which, through modulation of membrane potential, cell volume, and ion concentration promotes the movement of microglial processes and the initiation of phagocytosis. However, it is not known whether these mechanisms are important for aberrant nociceptive processing under pathological pain conditions.

Recently, a family of ion channels has been identified belonging to the TMEM16 family of proteins, which display functional diversity and may contribute to microglial function. The founding member of this family TMEM16A acts as a Ca^{2+}-activated Cl^- channel (Caputo et al., 2008; Schroeder et al., 2008; Yang et al., 2008), and another member TMEM16F has been proposed to be a Ca^{2+}-dependent phospholipid scramblase (Suzuki et al., 2010), and a Ca^{2+}-activated channel with either anion (Almaça et al., 2009; Martins et al., 2011; Tian et al., 2012) or non-selective cation (Yang et al., 2012) permeability. Each of these molecular mechanisms could contribute to spinal microglial function in neuropathic pain states.

To investigate whether TMEM16 channels play a role in neuropathic pain, we assayed the expression levels of all TMEM16 family members in microglia and observed high enrichment of

 © 2016 The Authors.
This is an open access article under the CC BY license (http://creativecommons.org/licenses/by/4.0/).
CrossMark

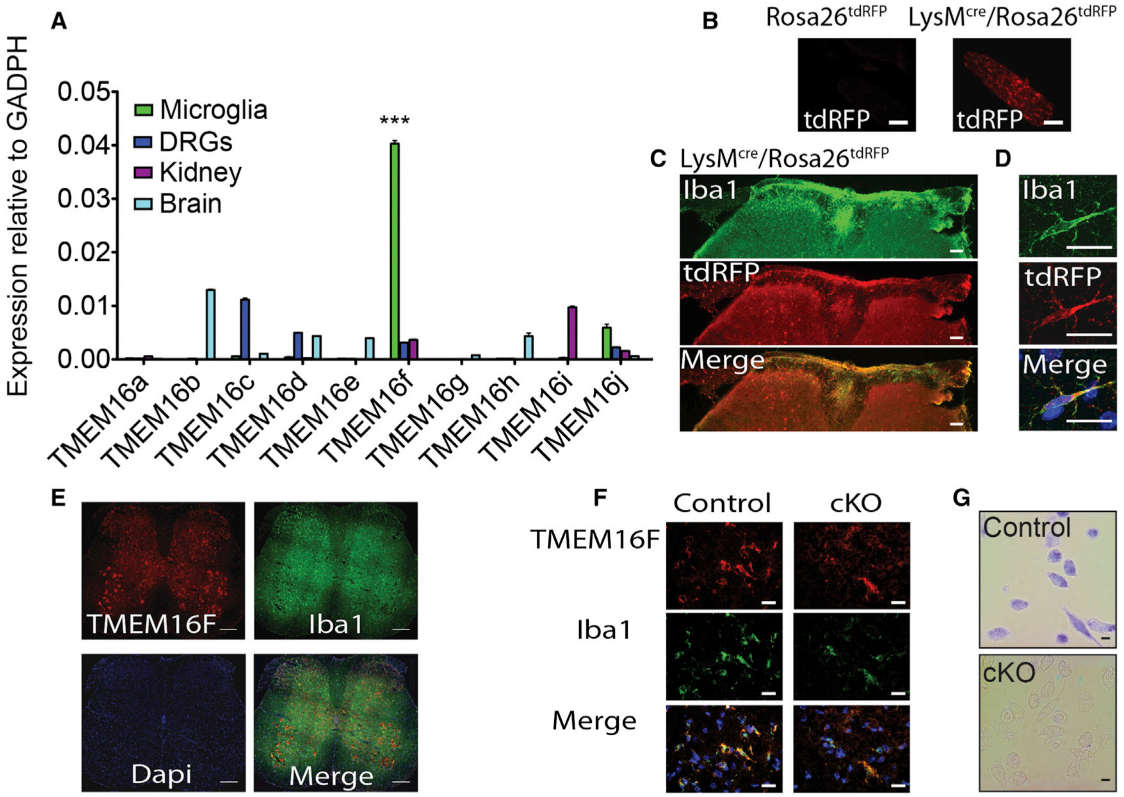

Figure 1. TMEM16 Expression and Generation of Conditional TMEM16F Knockout Mice

(A) RT-PCR analysis of TMEM16 transcripts in microglia, dorsal root ganglia (DRGs), kidney, and brain; $p < 0.0001$; $n = 3$.
(B) Representative images of injured sciatic nerve from indicated genotypes, 7 days after injury.
(C) Iba1 (green) and RFP (red) immunofluorescence in the lumbar spinal cord *LysM^{Cre}/Rosa26^{tdRFP}* mouse, 3 days after injury.
(D) Iba1 (green) and RFP (red) immunofluorescence of microglia in the hippocampus from *LysM^{Cre}/Rosa26^{tdRFP}* mouse.
(E) In situ hybridization for TMEM16F (red) and Iba1 (green) immunofluorescence of microglia in spinal cord, 7 days after injury.
(F) High-magnification representative images of TMEM16F in situ hybridization in control and cKO mice.
(G) In situ hybridization for TMEM16F in peritoneal macrophages cells from control and cKO mice.
Values are mean ± SEM. Scale bars, 100 and 40 μm (B), 100 μm (C), 20 μm (D, F, and G), 300 μm (E). See also Figure S1.

TMEM16F transcript. We thus generated a conditional knockout mouse line in which TMEM16F is genetically ablated in cells of the myeloid lineage. We demonstrate that, in the absence of TMEM16F, microglia are dysmorphic and exhibit deficits in process motility and phagocytosis. Moreover, TMEM16F conditional knockout mice display a pronounced reduction in mechanical hypersensitivity after peripheral nerve injury, suggesting that the classical scavenger function of microglia could be a factor in the pathogenesis of neuropathic pain.

RESULTS

TMEM16 Expression Profiling and Generation of a TMEM16F Conditional Knockout Mouse Line

In order to identify TMEM16 proteins with a potential role in pain processing, we performed expression profiling of all ten TMEM16 family members in dorsal root ganglia (DRG), microglia, and brain and compared their relative expression to basal levels in kidney using qRT-PCR. We observed a strikingly high expression of TMEM16F in microglia that was not apparent in other tissues tested (Figure 1A). Moreover, TMEM16F is the predominant microglial TMEM16 transcript with levels 4-fold higher than the next highest transcript TMEM16J.

To investigate the significance of the high expression of TMEM16F in microglia, we generated conditional TMEM16F knockout mice (Figure S1A). We tested two separate Cre driver lines, *CX3CR1^{Cre}* (Yona et al., 2013) and *LysM^{Cre}* (Clausen et al., 1999), crossed with Cre-dependent reporter lines for selective microglial and macrophage recombination. As previously reported (Eriksson et al., 1993), we observed substantial microgliosis in ipsilateral spinal cord upon injury. However, in *CX3CR1^{Cre}::Rosa26^{tdRFP}* mice, reporter expression was evident in both microglia and neurons throughout the spinal cord (Figure S1E). In contrast, *LysM^{Cre}::Rosa26^{tdRFP}* mice displayed selective expression in macrophages recruited to the injury site, and in microglia in the spinal cord and brain (Figures 1B–1D). In situ hybridization for TMEM16F and immunostaining for microglial marker Iba1 in spinal cord sections from injured mice, confirmed robust TMEM16F expression in spinal microglia from *LysM^{Cre}::TMEM16F$^{fl/+}$* mice and a decrease in TMEM16F mRNA in

OPEN
ACCESS
CellPress

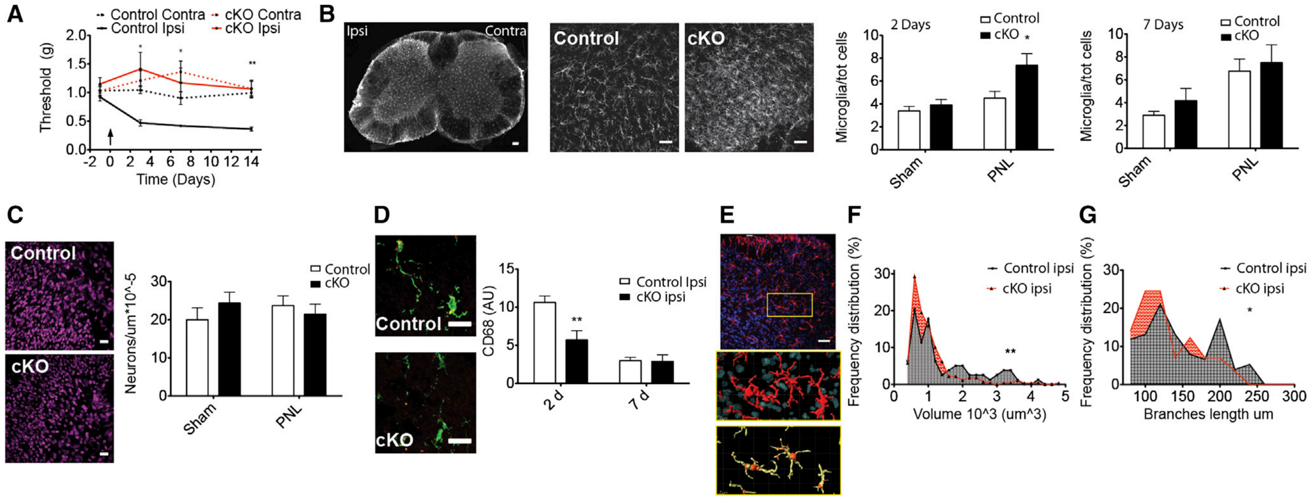

Figure 2. Microglial TMEM16F Is Required for the Development of Mechanical Hypersensitivity in the PNL Neuropathic Pain Model

(A) Paw withdrawal thresholds of *TMEM16F*$^{fl/fl}$ (control) and *LysM*^{cre}/*TMEM16F*$^{fl/fl}$ *(cKO)* mice showing ipsilateral (ipsi) and contralateral (contra) paw withdrawals before and after partial nerve ligation (PNL). n = 9; $p < 0.05$.

(B) From left to right: microglial Iba1 immunofluorescence in the lumbar spinal cord after injury (left side), magnified images from control and cKO tissues 2 days after PNL. Bar graphs show microglial densities in injured (PNL) and non-injured (sham) tissue 2 (left) and 7 (right) days after injury. n = 8/9; $p < 0.05$.

(C) Neuronal marker NeuN immunofluorescence in the ipsilateral dorsal horn from control (top) and cKO (bottom) mice; bar graph showing neuronal density in the imaged volume. n = 10/7; $p < 0.05$.

(D) CD68 (red) and Iba1 (green) immunofluorescence in the ipsilateral dorsal horn from control and cKO mice. CD68 immunoreactivity in Iba1 positive cells at 2 and 7 days after injury. n = 6; $p < 0.01$.

(E) Top: Iba1 (red) and DAPI (blue) immunolabeling in the ipsilateral dorsal horn; 3D image segmentation using IMARIS Bitplane surface (middle) and filament (bottom) algorithms.

(F) Frequency distribution of microglial volume in the ipsilateral dorsal horn from control and cKO mice, n = 79/277 cells; p = 0.005.

(G) Frequency distribution of microglial total branch length in the ipsilateral dorsal horn from control and cKO mice; p = 0.0293.

Values are mean ± SEM. Scale bars represent 100 μm (B), 30 μm (C), 20 μm (D), 40 μm, and 7 μm (E). See also Figures S2 and S3.

microglia and macrophages from *LysM*^{Cre}::*TMEM16F*$^{fl/fl}$ mice (Figures 1E–1G). We thus performed all further analysis on the *LysM*^{Cre}:: *TMEM16F*$^{fl/fl}$ (cKO) line using *TMEM16F*$^{fl/fl}$ (in the absence of Cre) or *LysM*^{Cre}:: *TMEM16F*$^{fl/+}$ mice as controls.

Microglial TMEM16F Is Required for Neuropathic Pain Development

To determine whether TMEM16F contributes to microglial function in pain states, we monitored nociceptive behavior in *LysM*^{Cre}::*TMEM16F*$^{fl/fl}$ mice in models of neuropathic and inflammatory pain. In control mice subjected to partial nerve ligation (PNL), we observed a pronounced mechanical allodynia that peaked at 7 days post-injury. Strikingly, deletion of TMEM16F in microglia and macrophages prevented the development of allodynia, and mechanical withdrawal thresholds remained at pre-injury levels throughout the monitoring period (Figure 2A), an effect that was observed in both male and female mice (Figure S2A).

To investigate the contribution of microglial TMEM16F to inflammatory pain, we used the Complete Freund's Adjuvant (CFA) model. We observed mechanical allodynia 48 hr after intraplantar injection of CFA in control animals. In contrast to the PNL model, *LysM*^{Cre}::*TMEM16F*$^{fl/fl}$ mice developed mechanical hypersensitivity to the same extent as litter mate controls (Figure S2B).

To explore whether TMEM16F contributes to proliferative, migratory, and phagocytic responses of microglia upon peripheral nerve injury, we performed quantitative image analysis on immunohistologically labeled spinal cord sections. Intriguingly, the total number of Iba1 positive microglia increased more rapidly after injury in lumbar spinal cords from *LysM*^{Cre}:: *TMEM16F*$^{fl/fl}$ compared to control mice, and 3 days post-injury this had already reached maximal levels. In contrast, microglial density from control mice required 7 days to reach similar levels (Figure 2B). We observed no difference in the number of neuronal cell bodies in at seven days post-injury between control and conditional knockout (cKO) animals (Figure 2C).

We investigated the activation status of microglia by quantifying lysosomal protein CD68 immunoreactivity (Holness and Simmons, 1993) in Iba1 positive cells and observed a dramatic increase at 2 days post-injury in control spinal cords that had normalized to baseline levels 7 days post-injury. In contrast, in spinal cords from *LysM*^{Cre}::*TMEM16F*$^{fl/fl}$ mice, CD68 immunoreactivity was significantly reduced compared to control (Figure 2D), suggesting that microglial phagocytic activity was impaired in the absence of TMEM16F.

We further examined morphological characteristics of spinal microglia, using 3D reconstruction of spinal cord confocal stacks and segmentation of Iba1 positive cells (Figure 2E). Microglia were significantly smaller in size and less branched in the ipsilateral dorsal horn of cKO mice compared to controls (Figures 2F and 2G).

As an additional measure of microglial function, we performed immunostaining for P2X4 receptors. Upon PNL, there was an

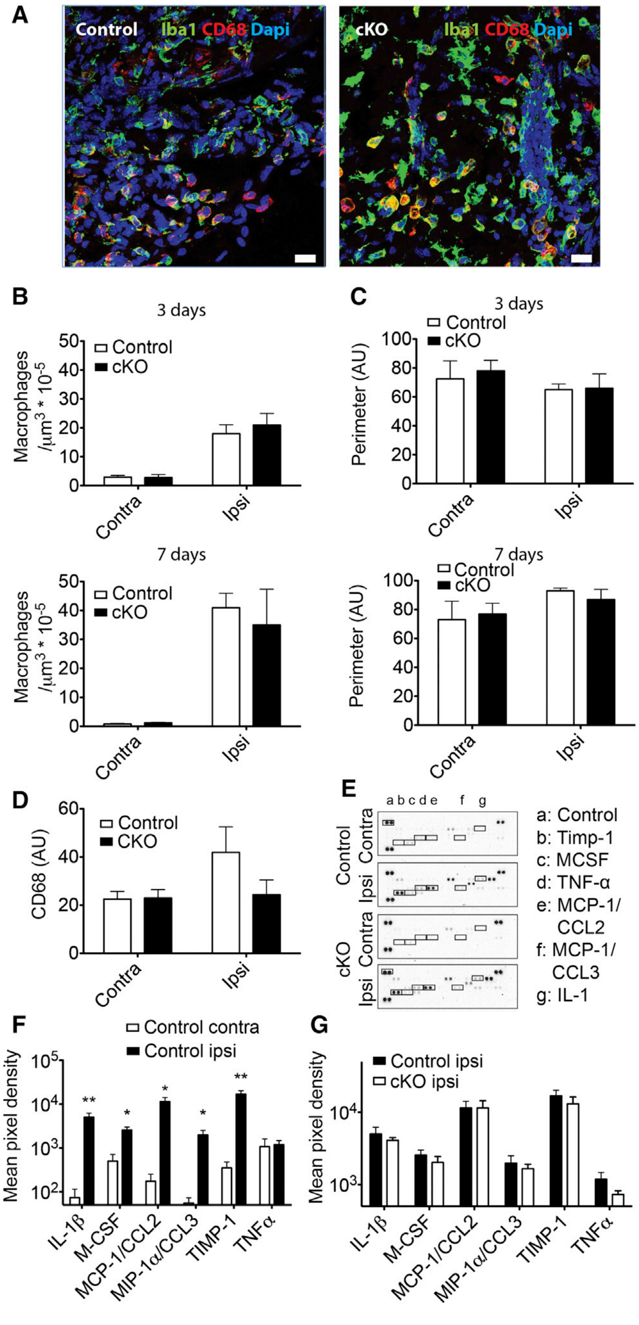

Figure 3. Histological and Biochemical Analysis of Macrophages Recruited at the Nerve Injury

(A) Iba1 (red), CD68 (green), and DAPI (blue) immunofluorescence in the ipsilateral nerve cryosections from control and cKO mice, 7 days after injury. Scale bar, 20 μm.

(B) Density of Iba1 positive macrophages at the distal side of the nerve injury at 3 and 7 days after injury. n = 3, $p < 0.05$.

(C) Size of Iba1 positive macrophages on the distal side of the nerve injury at 3 and 7 days after injury; n = 3; $p < 0.05$.

(D) CD68 intensity in Iba1 positive macrophages, 7 days after PNL; n = 3; $p > 0.05$.

(E) Cytokine/chemokine array blots incubated with contralateral and ipsilateral sciatic nerve extracts (130 μg) from control and cKO mice, 4 days after injury.

(F) Pixel densities of selected cytokine spots in the contralateral and ipsilateral sciatic nerve lysates from control mice. $p < 0.05$, $p < 0.01$. n = 3 sets of pooled samples from four mice each.

(G) Pixel densities of selected cytokine spots in the ipsilateral sciatic nerve lysates from control and cKO mice 4 days after injury, $p > 0.05$.

Values are mean ± SEM.

increase in P2X4 staining in the ipsilateral side compared to contralateral (73% ± 16%), also observed in the cKO mice (92% ± 17%; Figure S3).

To determine whether genetic ablation of TMEM16F perturbed the function of macrophages in the PNL model, we assessed macrophage recruitment, morphology, and activation at the nerve injury site (Figure 3A). In contrast to data from microglia, we observed no significant difference in the number (Figure 3B), size (Figure 3C), or CD68 immunoreactivity (Figure 3D) of macrophages in cKO mice compared to controls. Moreover, release of pro-inflammatory cytokines and chemokines upon nerve injury was also not significantly changed in $LysM^{Cre}$::$TMEM16F^{fl/fl}$ mice (Figures 3E–3G). Thus, TMEM16F may play a more prominent role in microglia compared to peripheral macrophages in the PNL model of neuropathic pain.

TMEM16F Influences Microglia Motility and Engulfment of Neuronal Material

We developed an ex vivo preparation for live imaging of microglia, using a triple transgenic $CX3CR1^{GFP}$:: $LysM^{Cre}$:: $TMEM16F^{fl/fl}$, in which GFP is robustly expressed in microglia.

We examined microglial branch extension in hippocampal slices by applying an ATP puff through a glass micropipette to mimic ATP release from damaged neurons (Davalos et al., 2005). Strikingly, the directed movement of microglia branches was significantly reduced in hippocampal slices from cKO^{GFP} compared to $Control^{GFP}$ mice (Figure 4A; Movie S1). Using tracking analysis of single microglial branches, we observed that the mean elongation velocity of single tracks after an ATP puff was significantly reduced in cKO^{GFP} (1.11 ± 0.03 μm/min) compared to $Control^{GFP}$ mice (1.28 ± 0.06 μm/min; $p = 0.017$, t test).

As an additional marker of peripheral sensory input into the spinal cord, Cholera Toxin B (CTB)-Alexa 647 was injected intraneurally into the sciatic nerve when performing PNL surgery. During live imaging from spinal cord slices, microglia from cKO^{GFP} mice displayed significantly reduced motility (Figure 4B; Movie S2). Strikingly, we observed that the engulfment of CTB-Alexa-647-labeled nerve terminals was reduced in cKO^{GFP} compared to $Control^{GFP}$ mice (Figure 4C; Movie S3).

In agreement with observations from ex vivo spinal cord preparations, isolated microglia from $Rosa26^{Cl-sensor}$::$LysM^{Cre}$:: $TMEM16F^{fl/fl}$ ($cKO^{Cl-sensor}$) mice displayed reduced phagocytosis of fluorescently labeled yeast, compared to control $Rosa26^{Cl-sensor}$:: $LysM^{Cre}$::$TMEM16F^{fl/+}$ (1.6 ± 0.2, n = 96 for control versus 0.9 ± 0.1, n = 87 for cKO, $p < 0.05$, t test, at 30 min) (Movie S4).

What is the link between impaired microglial phagocytosis and reduced neuropathic pain in TMEM16F cKO mice? Intriguingly, sciatic nerve injury has previously been associated with a

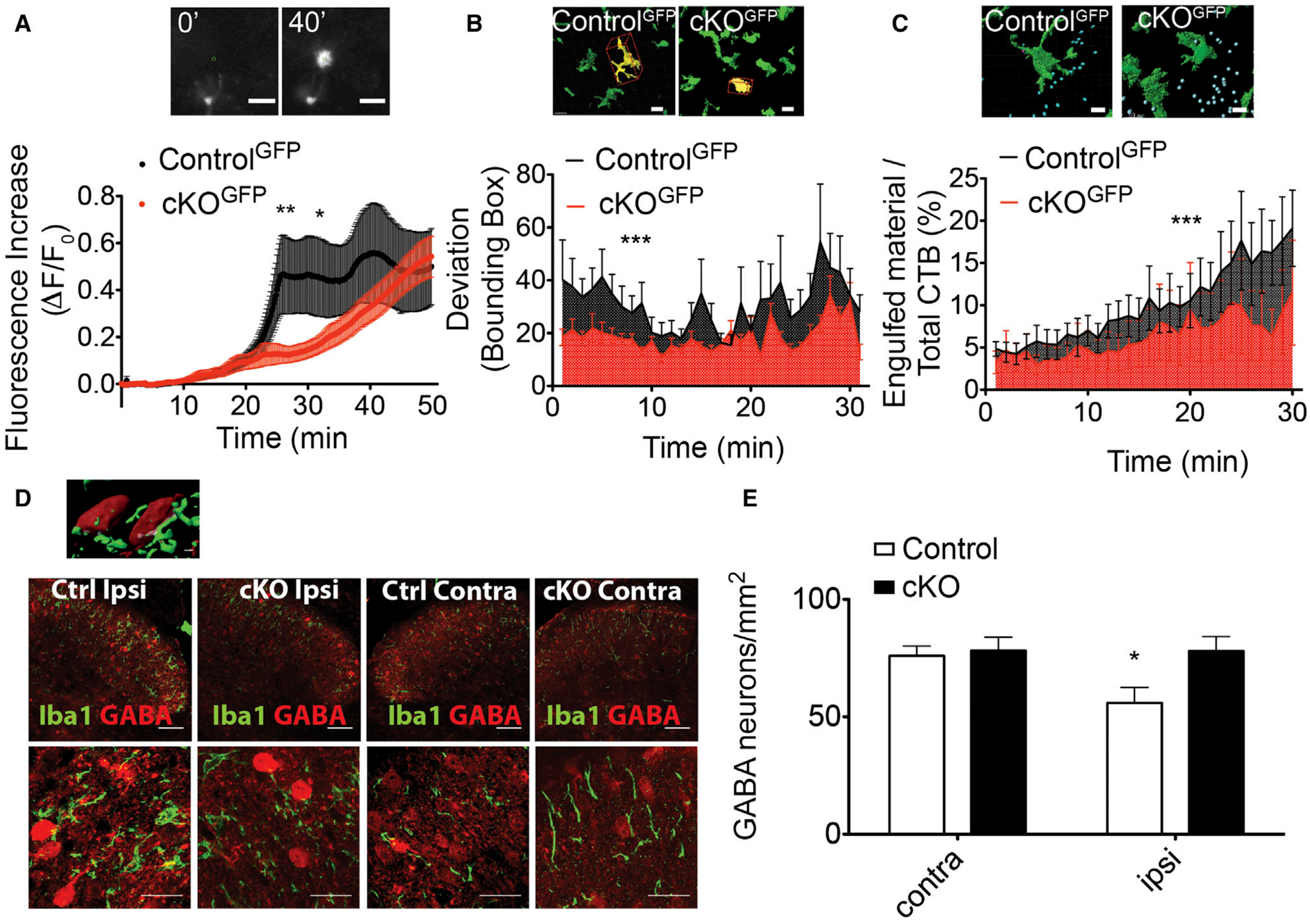

Figure 4. TMEM16F Regulates Microglia Branch Motility and Engulfment of Neuronal Material

(A) Top: fluorescence changes in a Control^{GFP} hippocampal slice near the tip (green squares). Bottom: relative fluorescence increase measured in a 10-μm radius from the ATP-containing (3 mM) pipette in acute hippocampal slices from mice of indicated genotypes, n = 9/24 fields in three of seven mice; $p < 0.05$ from 25 to 34 min.

(B) Top: ipsilateral dorsal horn sections from mice of indicated genotypes. The selected segmented GFP positive microglia (yellow) is surrounded by a bounding box (red), which was used for branch motility analysis. Bottom: SD of the bounding box from its mean value over time to quantify microglia branch motility in spinal cord slices, from mice of indicated genotypes.; n = 44/45 cells; $p < 0.0001$.

(C) Segmentation of GFP positive microglia (green) and Alexa-647-labeled neurons, external to microglia (blue) and internalized (pink). Ratio of internalized over total labeled neuronal material to quantify engulfed neuronal terminals by microglia over time.

(D) Top: image segmentation: GABA positive neuron (red) colocalizes (white) within Iba1 positive microglia (green) after injury. Bottom: Iba1 (green) and GABA (red) immunofluorescence in the ipsilateral and contralateral dorsal horns from control and cKO mice, 3 days after injury, with zoomed images from lamina I–III in the insets.

(E) Density of GABA-positive neurons in the lamina I–III of the dorsal horn of control and cKO mice, 3 days after injury. $p < 0.05$, n = 5.

Values are mean ± SEM. Scale bar, 50 μm (B and C) and 60 μm (D). See Movies S1, S2, S3, and S4.

selective loss of GABA in the ipsilateral dorsal horn, which, in turn, reduces inhibitory control in the spinal cord and promotes mechanical hypersensitivity (Scholz et al., 2005; Sugimoto et al., 1990). We therefore asked whether loss of GABA might be spared in TMEM16F cKO mice, perhaps as a consequence of the diminished phagocytic capacity of microglia. Utilizing immunohistochemistry for GABA in lumbar spinal cord sections, we observed a significant reduction in GABA staining in the ipsilateral dorsal horn of control mice upon injury (Figure 4D). Strikingly, in TMEM16F knockout mice this reduction was not apparent, and ipsilateral and contralateral sides displayed similar numbers of GABA positive neuron (Figure 4E).

DISCUSSION

In this study, we explored the significance of high expression levels of TMEM16F in microglia via conditional ablation of the TMEM16F gene in cells of the myeloid lineage. We demonstrate that TMEM16F conditional knockout mice do not develop mechanical hypersensitivity after peripheral nerve injury and that this is associated with a reduced phagocytosis by spinal microglia. Moreover, in both in vivo and ex vivo preparations, microglia exhibit deficits in function that is reflected in their altered morphology, expression of activation markers, and diminished branch motility and phagocytic capacity. Together our data

suggest that that the phagocytic activity of microglia is an important component in the cascade of events that lead to altered nociceptive processing in neuropathic pain.

We opted for a conditional genetic strategy to delete TMEM16F and after assessing two Cre driver lines (Clausen et al., 1999; Yona et al., 2013) for reporter gene expression, selected *LysM*^{Cre} as this drove expression specifically in microglia in the spinal cord. However, Cre-mediated recombination was not evident in all microglia. We addressed this issue in in vitro studies by using triple transgenic mice expressing Cre-dependent reporter and only selecting fluorescent cells for analysis. In in vivo and ex vivo studies, however, the incomplete deletion of TMEM16F from all microglia will presumably lead to an underestimation of phenotypes. A further complication arising from use of the *LysM*^{Cre} driver lines is that recombination will also occur in macrophages. We observed no differences in macrophage number, morphology, or activation at the injury site in the sciatic nerve in cKO versus control mice, or in the expression of inflammatory mediators in the injured nerve. Moreover, in the CFA inflammatory pain model, which does not provoke microglial activation (Li et al., 2013; Lin et al., 2007), nociceptive thresholds were similar between genotypes. These data imply that TMEM16F has a more prominent role in microglia than macrophages in neuropathic pain models. The further development of Cre-driver lines, which selectively target microglia with high efficiency, for example, inducible *Cx3cr1*^{CreER} mice (Parkhurst et al., 2013), will allow for a more direct assessment of these issues.

Upon peripheral nerve injury, microglia migrate to the spinal cord, proliferate, and assume an activated state (Gehrmann et al., 1991; Guan et al., 2016). This is accompanied by an increase in P2X4 receptor expression, release of BDNF, and disinhibition of lamina I neurons (Coull et al., 2003, 2005). We therefore asked whether deletion of TMEM16F in microglia would impact upon any of these mechanisms. We observed no decrease in microglial recruitment to the spinal cord (indeed this was significantly increased in TMEM16F cKO mice), and upregulation of P2X4 receptor immunoreactivity in the ipsilateral dorsal horn of TMEM16F cKO mice at levels similar to that seen in control mice.

Other factors have also been demonstrated to contribute to a loss of inhibition in the spinal cord, including a decrease in GABA production and release, and degeneration of GABAergic interneurons (Bráz et al., 2012; Moore et al., 2002; Scholz et al., 2005; Sugimoto et al., 1990). Intriguingly, we observed a reduction in GABA immunoreactivity in lamina I–III of the ipsilateral dorsal horn of control mice, which was absent in the cKO mice. Together with our data on the impaired phagocytic capacity of TMEM16F cKO microglia, we speculate that diminished phagocytosis may spare GABA loss and thus reduce disinhibition and the development of mechanical hypersensitivity. This mechanism could occur independently of complete loss of inhibitory neurons by necrosis or apoptosis and instead happen via selective pruning of GABAergic terminals. Indeed, recent work by Petitjean et al. (2015) has demonstrated that parvalbumin-positive interneurons do not die after peripheral nerve injury but exhibit reduced connectivity with PKCγ neurons in the dorsal horn. Thus, in addition to the well-established role of microglia in releasing bioactive factors (Clark et al., 2007, 2013), their classical phagocytosis function may also contribute to the pathogenesis of neuropathic pain. Further investigation of the integrity of spinal pain circuitry in TMEM16F cKO mice, as well as its role in other cell types (Jiang et al., 2016; Sorge et al., 2015), will shed more light on this issue.

TMEM16F functions as both a calcium dependent ion channel and a phospholipid scramblase. Therefore, an important question that arises from our data is whether deficits in microglial phagocytosis are caused by alterations in ion transport or in phosphatidylserine exposure in cKO cells. Intriguingly, both processes have previously been shown to be important for phagocytosis and ramification in macrophages and microglia suggesting that TMEM16F could utilize multiple mechanisms to modulate microglial function (Callahan et al., 2000; Eder et al., 1998). Recently, TMEM16F was also demonstrated to act downstream of P2X7 receptors and influence immune defense by macrophages (Ousingsawat et al., 2015). While we observed no deficits in macrophage function upon nerve injury in TMEM16F knockout mice, other purinergic receptors such as P2Y12 receptors have been implicated in microglial phagocytosis. Indeed, P2Y12 is expressed exclusively by microglia (Hickman et al., 2013; Kobayashi et al., 2011) and has been demonstrated to play a key role in ATP-mediated branch rearrangement and phagocytosis of injured axons (Haynes et al., 2006; Maeda et al., 2010; Ohsawa et al., 2010). Investigation of interactions between TMEM16F and P2Y12 could therefore be a useful starting point for developing therapeutic strategies that target microglia in neuropathic pain states.

EXPERIMENTAL PROCEDURES

Details are further described in Supplemental Experimental Procedures.

Animals

Mice were bred and maintained at the EMBL Mouse Biology Unit, Monterotondo, in accordance with Italian legislation under license from the Italian Ministry of Health. The TMEM16F targeting strategy was designed to allow Cre-mediated excision of the exons 13 of the TMEM16F, resulting in a frameshift mutation in exon 14. The *LysM*^{Cre} line (Clausen et al., 1999) was used as a driver line for conditional ablation of TMEM16F. Crosses with *Rosa26*^{tdRFP}, *Rosa26*$^{Cl-sensor}$, and *CX3CR1*^{GFP} mouse lines yielded reporters for visual and functional tracking.

Pain Models and Behavioral Assays

Partial nerve ligation of the left sciatic nerve was performed on mice of both sexes to induce neuropathic pain behavior as described previously (Seltzer et al., 1990). Mice were tested blindly for mechanical allodynia using calibrated von Frey filaments of increasing force applied to the hindpaw and fitting the paw withdrawal probability. Inflammatory pain was induced by intraplantar injection of Complete Freund's Adjuvant (CFA). To trace central sensory endings, 2-μl injections of 0.5% cholera Toxin-B (CTB) Alexa Fluor 647 conjugate were performed into the sciatic nerve.

Double RNA Fluorescent In Situ Hybridization and Immunofluorescence

In situ hybridization (ISH) was performed on spinal cord cryosections using a fluorescein-labeled probe generated from a full-length TMEM16F cDNA. Briefly, sections were fixed in 4% paraformaldehyde (PFA), digested with proteinase K for 5 min, acetylated, and hybridized with the probes in 50% formamide, 5 × saline sodium citrate (SSC), 5 × Denhardt's solution, 500 μg/ml salmon sperm DNA, and 250 μg/ml tRNA overnight at 56°C. After stringent

post-hybridization washes, sections were blocked and incubated with mouse anti-fluorescein (Roche; at 1:100) and rabbit anti-Iba1 (Wako; at 1:200), followed by anti-mouse Alexa 555 and anti-rabbit Alexa 488.

Real-Time PCR

Homogenates of DRG, brain, kidney, L4–L6 segment of the lumbar spinal cord, and lysates of microglia cells were subjected to total RNA extraction and qPCR according to standard protocol. Each mRNA expression level was normalized to ubiquitin or GADPH.

Western Blotting

Harvested tissues were homogenized on ice in lysis buffer proteinase inhibitor. 10-μg lysates were loaded for gel electrophoresis. Western blot was performed using standard techniques with rabbit anti-TMEM16F (HPA038958, Sigma).

Immunohistochemistry

Immunohistochemistry was performed on paraformaldehyde-fixed cryosections and free floating sections according to standard protocols. The following primary antibodies were incubated overnight at 4°C: rabbit anti-Iba1 (019-19741, Wako; 2.5 μg/ml), rat anti-CD68 (Abd Biotech; 10 μg/ml), mouse anti-NeuN (1:250), rabbit anti-P2X4 receptor (ab82329, Abcam; 1:200), rabbit anti-RFP (600-401-379, Rockland; 5 μg/ml), and rabbit anti-GABA (A2052, Sigma;1:2,000). For co-staining with RFP or P2X4R or GABA, a mouse goat anti-Iba1 (Novus; 5 μg/ml) was used. Anti-rabbit-, anti-rat, and anti-mouse Alexa 488, 546, or 647 secondary antibodies (2 μg/ml) (Life Technologies) were used. *Z* optical series covered 42 μm of thickness for free floating sections and 12 μm for cryosections with 0.5 μm step.

Time-Lapse Microscopy of Microglia

Time-lapse imaging of microglia in the culture and spinal cord slices was carried out on a spinning-disk confocal ultraview Vox (Cellular imaging, PerkinElmer) at 37°C and 5% CO_2. For spinal cord, ex vivo time-lapse *Z* optical series covered 40 μm of thickness with 0.5 μm step. For phagocytosis assays, primary microglia cells expressing endogenous Cl^-Sensor (Batti et al., 2013) were co-incubated with fluorescent heat-killed *S. cerevisiae* yeast. Images were taken every minute for 30 min.

Time-lapse imaging of microglial branch extension in hippocampal slice from *CX3CR1^GFP^* mice was performed at room temperature during 2–7 hr after cutting. Adenosine 5′-triphosphate magnesium salt (ATP, 2 mM; Sigma-Aldrich) was pressure applied (100 ms; 5 psi) from the glass pipette placed in the stratum radiatum. GFP fluorescence was measured every 10 s for 50 min in a 20-μm diameter area around the pipette tip to quantify the speed of GFP-expressing microglial processes extension.

Image Analysis

Three-dimensional reconstructions of confocal stacks and surface rendering were performed with Imaris Bitplane Software (Bitplane). Surface, Filament, and Spots Imaris modules were used for segmentation and tracking of microglial cells, microglia processes, and neuron terminals, respectively. Tracking analysis of single microglial processes in the hippocampal slice was performed using ImageJ software.

Cytokine Arrays

Protein extracts from sciatic nerve fragments were processed with pre-spotted cytokine/chemokine array according to manufacturer's instructions (R&D Systems, mouse cytokine array panel A, no. ARY006). Signal intensity was analyzed in ImageJ, background subtracted, and averaged on duplicates and between three sets of four mice.

Statistical Analysis

Statistical significance was determined as $p < 0.05$ by one-way ANOVA (Figures 2A, 3D, 3F, 3G, and 4C) or two-way ANOVA test (Figures 1A, 2B–2D, 3B, 3C, and 4B) followed by Bonferroni post hoc test, Student's t test (Figure 4A), χ^2 test, or Mann Whitney test (Figures 2F and 2G).

SUPPLEMENTAL INFORMATION

Supplemental Information includes Supplemental Results, Supplemental Experimental Procedures, three figures, and four movies and can be found with this article online at http://dx.doi.org/10.1016/j.celrep.2016.05.039.

AUTHOR CONTRIBUTIONS

L.B. and M.S. contributed equally to this work. L.B., M.S., E.M., F.P., and P.A.H. designed experiments. E.M., F.P., and M.S. performed functional in vitro and ex vivo studies. L.B. performed imaging and segmentation analysis. L.B., S.P., and M.S. performed behavioral tests and surgery. L.B., S.P., and M.S. performed biochemistry and immunohistochemistry. F.D.C.R. performed southern blot and RT-PCR. H.W. generated the mouse. E.P. performed tracking analysis. L.B., M.S., P.A.H., F.P., S.D.A., and D.R. supervised the project, L.B., M.S., and P.A.H. co-wrote the manuscript.

ACKNOWLEDGMENTS

We thank EMBL Monterotondo Transgenic facility, Mouse Phenotyping facility, Histology facility, and Microscopy facility, Violetta Parimbeni and Matteo Gaetani for mouse husbandry, Maurizio Abbate for image analysis advice, and Carsten Schultz for helpful discussions. We gratefully acknowledge the Seventh Framework Programme Intra-European Fellowship (IEF) and a Young Investigator Programme fellowship from the Veronesi Foundation for support for L.B. and an Interdisciplinary Postdoctoral Fellowship (EIPOD, EMBL, and EU Marie Curie Actions COFUND II grant) for support for M.S.

Received: March 18, 2015
Revised: December 10, 2015
Accepted: May 7, 2016
Published: July 9, 2016

REFERENCES

Almaça, J., Tian, Y., Aldehni, F., Ousingsawat, J., Kongsuphol, P., Rock, J.R., Harfe, B.D., Schreiber, R., and Kunzelmann, K. (2009). TMEM16 proteins produce volume-regulated chloride currents that are reduced in mice lacking TMEM16A. J. Biol. Chem. *284*, 28571–28578.

Batti, L., Mukhtarov, M., Audero, E., Ivanov, A., Paolicelli, R.C., Zurborg, S., Gross, C., Bregestovski, P., and Heppenstall, P.A. (2013). Transgenic mouse lines for non-invasive ratiometric monitoring of intracellular chloride. Front. Mol. Neurosci. *6*, 11.

Beggs, S., Trang, T., and Salter, M.W. (2012). P2X4R+ microglia drive neuropathic pain. Nat. Neurosci. *15*, 1068–1073.

Bráz, J.M., Sharif-Naeini, R., Vogt, D., Kriegstein, A., Alvarez-Buylla, A., Rubenstein, J.L., and Basbaum, A.I. (2012). Forebrain GABAergic neuron precursors integrate into adult spinal cord and reduce injury-induced neuropathic pain. Neuron *74*, 663–675.

Callahan, M.K., Williamson, P., and Schlegel, R.A. (2000). Surface expression of phosphatidylserine on macrophages is required for phagocytosis of apoptotic thymocytes. Cell Death Differ. *7*, 645–653.

Campbell, J.N., and Meyer, R.A. (2006). Mechanisms of neuropathic pain. Neuron *52*, 77–92.

Caputo, A., Caci, E., Ferrera, L., Pedemonte, N., Barsanti, C., Sondo, E., Pfeffer, U., Ravazzolo, R., Zegarra-Moran, O., and Galietta, L.J. (2008). TMEM16A, a membrane protein associated with calcium-dependent chloride channel activity. Science *322*, 590–594.

Clark, A.K., Yip, P.K., Grist, J., Gentry, C., Staniland, A.A., Marchand, F., Dehvari, M., Wotherspoon, G., Winter, J., Ullah, J., et al. (2007). Inhibition of spinal microglial cathepsin S for the reversal of neuropathic pain. Proc. Natl. Acad. Sci. USA *104*, 10655–10660.

Clark, A.K., Old, E.A., and Malcangio, M. (2013). Neuropathic pain and cytokines: current perspectives. J. Pain Res. *6*, 803–814.

Clausen, B.E., Burkhardt, C., Reith, W., Renkawitz, R., and Förster, I. (1999). Conditional gene targeting in macrophages and granulocytes using LysMcre mice. Transgenic Res. *8*, 265–277.

Costigan, M., Scholz, J., and Woolf, C.J. (2009). Neuropathic pain: a maladaptive response of the nervous system to damage. Annu. Rev. Neurosci. *32*, 1–32.

Coull, J.A., Boudreau, D., Bachand, K., Prescott, S.A., Nault, F., Sík, A., De Koninck, P., and De Koninck, Y. (2003). Trans-synaptic shift in anion gradient in spinal lamina I neurons as a mechanism of neuropathic pain. Nature *424*, 938–942.

Coull, J.A., Beggs, S., Boudreau, D., Boivin, D., Tsuda, M., Inoue, K., Gravel, C., Salter, M.W., and De Koninck, Y. (2005). BDNF from microglia causes the shift in neuronal anion gradient underlying neuropathic pain. Nature *438*, 1017–1021.

Davalos, D., Grutzendler, J., Yang, G., Kim, J.V., Zuo, Y., Jung, S., Littman, D.R., Dustin, M.L., and Gan, W.B. (2005). ATP mediates rapid microglial response to local brain injury in vivo. Nat. Neurosci. *8*, 752–758.

Eder, C., Klee, R., and Heinemann, U. (1998). Involvement of stretch-activated Cl- channels in ramification of murine microglia. J. Neurosci. *18*, 7127–7137.

Eriksson, N.P., Persson, J.K., Svensson, M., Arvidsson, J., Molander, C., and Aldskogius, H. (1993). A quantitative analysis of the microglial cell reaction in central primary sensory projection territories following peripheral nerve injury in the adult rat. Exp. Brain Res. *96*, 19–27.

Fu, R., Shen, Q., Xu, P., Luo, J.J., and Tang, Y. (2014). Phagocytosis of microglia in the central nervous system diseases. Mol. Neurobiol. *49*, 1422–1434.

Gehrmann, J., Monaco, S., and Kreutzberg, G.W. (1991). Spinal cord microglial cells and DRG satellite cells rapidly respond to transection of the rat sciatic nerve. Restor. Neurol. Neurosci. *2*, 181–198.

Guan, Z., Kuhn, J.A., Wang, X., Colquitt, B., Solorzano, C., Vaman, S., Guan, A.K., Evans-Reinsch, Z., Braz, J., Devor, M., et al. (2016). Injured sensory neuron-derived CSF1 induces microglial proliferation and DAP12-dependent pain. Nat. Neurosci. *19*, 94–101.

Haynes, S.E., Hollopeter, G., Yang, G., Kurpius, D., Dailey, M.E., Gan, W.B., and Julius, D. (2006). The P2Y12 receptor regulates microglial activation by extracellular nucleotides. Nat. Neurosci. *9*, 1512–1519.

Hickman, S.E., Kingery, N.D., Ohsumi, T.K., Borowsky, M.L., Wang, L.C., Means, T.K., and El Khoury, J. (2013). The microglial sensome revealed by direct RNA sequencing. Nat. Neurosci. *16*, 1896–1905.

Holness, C.L., and Simmons, D.L. (1993). Molecular cloning of CD68, a human macrophage marker related to lysosomal glycoproteins. Blood *81*, 1607–1613.

Jiang, B.C., Cao, D.L., Zhang, X., Zhang, Z.J., He, L.N., Li, C.H., Zhang, W.W., Wu, X.B., Berta, T., Ji, R.R., and Gao, Y.J. (2016). CXCL13 drives spinal astrocyte activation and neuropathic pain via CXCR5. J. Clin. Invest. *126*, 745–761.

Kobayashi, K., Takahashi, E., Miyagawa, Y., Yamanaka, H., and Noguchi, K. (2011). Induction of the P2X7 receptor in spinal microglia in a neuropathic pain model. Neurosci. Lett. *504*, 57–61.

Lever, I., Cunningham, J., Grist, J., Yip, P.K., and Malcangio, M. (2003). Release of BDNF and GABA in the dorsal horn of neuropathic rats. Eur. J. Neurosci. *18*, 1169–1174.

Li, K., Tan, Y.H., Light, A.R., and Fu, K.Y. (2013). Different peripheral tissue injury induces differential phenotypic changes of spinal activated microglia. Clin. Dev. Immunol. *2013*, 901420.

Lin, T., Li, K., Zhang, F.Y., Zhang, Z.K., Light, A.R., and Fu, K.Y. (2007). Dissociation of spinal microglia morphological activation and peripheral inflammation in inflammatory pain models. J. Neuroimmunol. *192*, 40–48.

Maeda, M., Tsuda, M., Tozaki-Saitoh, H., Inoue, K., and Kiyama, H. (2010). Nerve injury-activated microglia engulf myelinated axons in a P2Y12 signaling-dependent manner in the dorsal horn. Glia *58*, 1838–1846.

Martins, J.R., Faria, D., Kongsuphol, P., Reisch, B., Schreiber, R., and Kunzelmann, K. (2011). Anoctamin 6 is an essential component of the outwardly rectifying chloride channel. Proc. Natl. Acad. Sci. USA *108*, 18168–18172.

Moore, K.A., Kohno, T., Karchewski, L.A., Scholz, J., Baba, H., and Woolf, C.J. (2002). Partial peripheral nerve injury promotes a selective loss of GABAergic inhibition in the superficial dorsal horn of the spinal cord. J. Neurosci. *22*, 6724–6731.

Ohsawa, K., Irino, Y., Sanagi, T., Nakamura, Y., Suzuki, E., Inoue, K., and Kohsaka, S. (2010). P2Y12 receptor-mediated integrin-beta1 activation regulates microglial process extension induced by ATP. Glia *58*, 790–801.

Ousingsawat, J., Wanitchakool, P., Kmit, A., Romao, A.M., Jantarajit, W., Schreiber, R., and Kunzelmann, K. (2015). Anoctamin 6 mediates effects essential for innate immunity downstream of P2X7 receptors in macrophages. Nat. Commun. *6*, 6245.

Parkhurst, C.N., Yang, G., Ninan, I., Savas, J.N., Yates, J.R., 3rd, Lafaille, J.J., Hempstead, B.L., Littman, D.R., and Gan, W.B. (2013). Microglia promote learning-dependent synapse formation through brain-derived neurotrophic factor. Cell *155*, 1596–1609.

Petitjean, H., Pawlowski, S.A., Fraine, S.L., Sharif, B., Hamad, D., Fatima, T., Berg, J., Brown, C.M., Jan, L.Y., Ribeiro-da-Silva, A., et al. (2015). Dorsal Horn Parvalbumin Neurons Are Gate-Keepers of Touch-Evoked Pain after Nerve Injury. Cell Rep. *13*, 1246–1257.

Scholz, J., and Woolf, C.J. (2007). The neuropathic pain triad: neurons, immune cells and glia. Nat. Neurosci. *10*, 1361–1368.

Scholz, J., Broom, D.C., Youn, D.H., Mills, C.D., Kohno, T., Suter, M.R., Moore, K.A., Decosterd, I., Coggeshall, R.E., and Woolf, C.J. (2005). Blocking caspase activity prevents transsynaptic neuronal apoptosis and the loss of inhibition in lamina II of the dorsal horn after peripheral nerve injury. J. Neurosci. *25*, 7317–7323.

Schroeder, B.C., Cheng, T., Jan, Y.N., and Jan, L.Y. (2008). Expression cloning of TMEM16A as a calcium-activated chloride channel subunit. Cell *134*, 1019–1029.

Seltzer, Z., Dubner, R., and Shir, Y. (1990). A novel behavioral model of neuropathic pain disorders produced in rats by partial sciatic nerve injury. Pain *43*, 205–218.

Sierra, A., Abiega, O., Shahraz, A., and Neumann, H. (2013). Janus-faced microglia: beneficial and detrimental consequences of microglial phagocytosis. Front. Cell. Neurosci. *7*, 6.

Sorge, R.E., Mapplebeck, J.C., Rosen, S., Beggs, S., Taves, S., Alexander, J.K., Martin, L.J., Austin, J.S., Sotocinal, S.G., Chen, D., et al. (2015). Different immune cells mediate mechanical pain hypersensitivity in male and female mice. Nat. Neurosci. *18*, 1081–1083.

Sugimoto, T., Bennett, G.J., and Kajander, K.C. (1990). Transsynaptic degeneration in the superficial dorsal horn after sciatic nerve injury: effects of a chronic constriction injury, transection, and strychnine. Pain *42*, 205–213.

Suzuki, J., Umeda, M., Sims, P.J., and Nagata, S. (2010). Calcium-dependent phospholipid scrambling by TMEM16F. Nature *468*, 834–838.

Tian, Y., Schreiber, R., and Kunzelmann, K. (2012). Anoctamins are a family of Ca2+-activated Cl- channels. J. Cell Sci. *125*, 4991–4998.

Tsuda, M., Shigemoto-Mogami, Y., Koizumi, S., Mizokoshi, A., Kohsaka, S., Salter, M.W., and Inoue, K. (2003). P2X4 receptors induced in spinal microglia gate tactile allodynia after nerve injury. Nature *424*, 778–783.

Yang, Y.D., Cho, H., Koo, J.Y., Tak, M.H., Cho, Y., Shim, W.S., Park, S.P., Lee, J., Lee, B., Kim, B.M., et al. (2008). TMEM16A confers receptor-activated calcium-dependent chloride conductance. Nature *455*, 1210–1215.

Yang, H., Kim, A., David, T., Palmer, D., Jin, T., Tien, J., Huang, F., Cheng, T., Coughlin, S.R., Jan, Y.N., and Jan, L.Y. (2012). TMEM16F forms a Ca2+-activated cation channel required for lipid scrambling in platelets during blood coagulation. Cell *151*, 111–122.

Yona, S., Kim, K.W., Wolf, Y., Mildner, A., Varol, D., Breker, M., Strauss-Ayali, D., Viukov, S., Guilliams, M., Misharin, A., et al. (2013). Fate mapping reveals origins and dynamics of monocytes and tissue macrophages under homeostasis. Immunity *38*, 79–91.

Cell Reports, Volume *15*

Supplemental Information

TMEM16F Regulates Spinal Microglial Function in Neuropathic Pain States

Laura Batti, Mayya Sundukova, Emanuele Murana, Sofia Pimpinella, Fernanda De Castro Reis, Francesca Pagani, Hong Wang, Eloisa Pellegrino, Emerald Perlas, Silvia Di Angelantonio, Davide Ragozzino, and Paul A. Heppenstall

TMEM16F regulates spinal microglial function in neuropathic pain states

Laura Batti[#], Mayya Sundukova[#], Emanuele Murana, Sofia Pimpinella, Fernanda De Castro Reis, Francesca Pagani, Hong Wang, Eloisa Pellegrino, Emerald Perlas, Silvia Di Angelantonio, Davide Ragozzino, Paul A. Heppenstall

[#]Co-first author

Supplemental Results, related to Fig.1

We designed a targeting construct whereby exon 13 of the TMEM16F gene was flanked by *loxp* sites, allowing for excision of the exon by Cre-mediated recombination, and a subsequent frame-shift mutation in exon 14 (mimicking a human mutation observed in Scott syndrome patients (Suzuki et al., 2010). A *frt* flanked neomycin cassette was introduced to enable selection of correctly targeted ES cells. Positive ES cell clones were identified using southern hybridization, and chimeric mice were obtained that were crossed with Flp-expressing transgenic mice to remove the neomycin cassette.

We first generated ubiquitous *TMEM16F* knockout mice (*TMEM16F*$^{-/-}$) by crossing mice with the *Deleter*^{Cre} line (Schwenk et al., 1995). Further crosses were undertaken to remove the Cre transgene and validation of TMEM16F ablation was achieved using southern hybridization of genomic DNA and western blotting from membrane fraction of spleen homogenates (**Fig. S1B,C**). From crosses of *TMEM16F*$^{+/-}$ mice we observed a reduction in the number of *TMEM16F*$^{-/-}$ offspring compared to expected Mendelian ratios (**Fig.S1D**). Moreover, *TMEM16F*$^{-/-}$ mice had reduced body weight in

comparison to their +/+ or +/- littermates (median value: -10% weight difference, n=6; $p > 0.005$). We thus opted for a conditional approach to target TMEM16F in microglia.

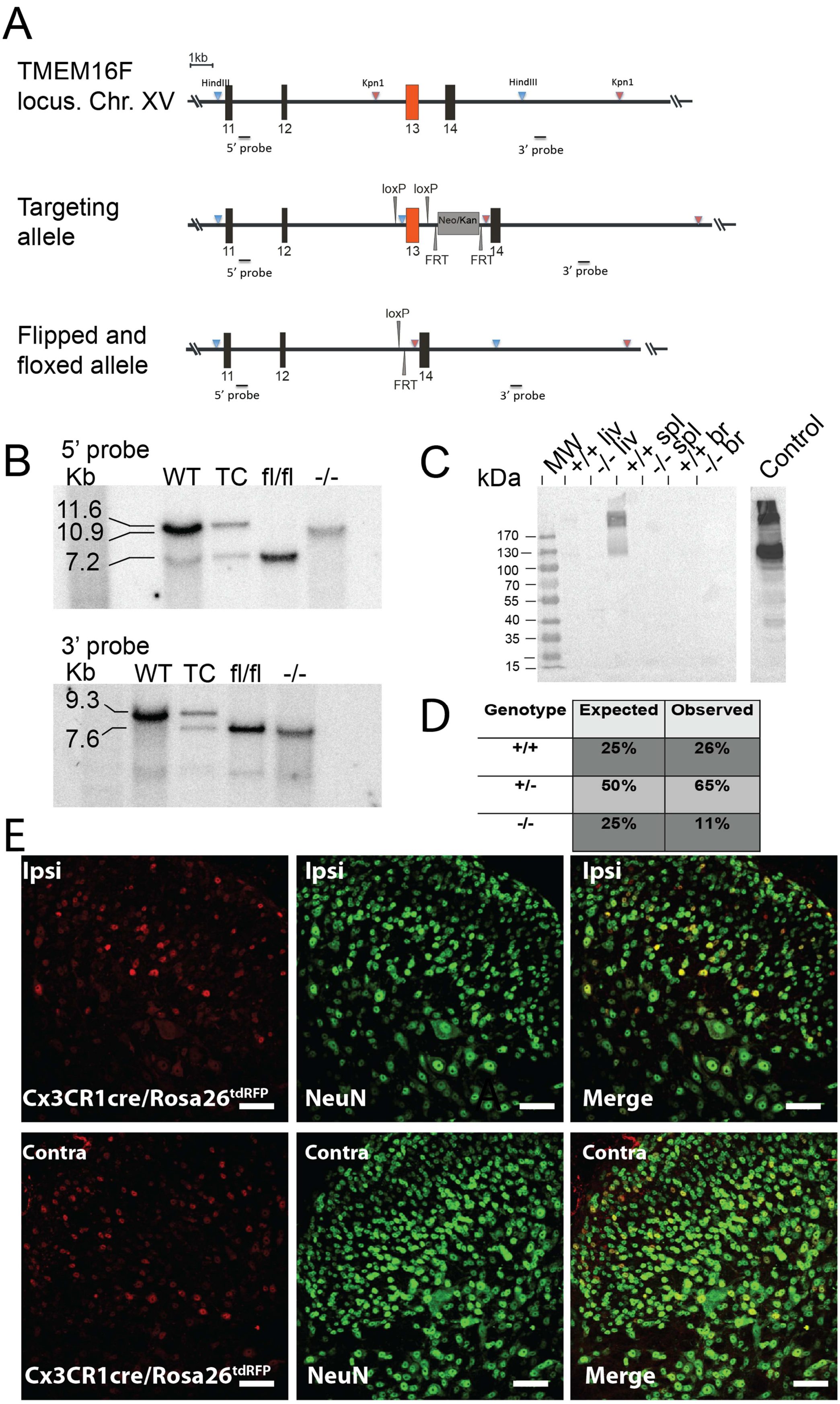

Genotype	Expected	Observed
+/+	25%	26%
+/-	50%	65%
-/-	25%	11%

Figure S1, related to Fig. 1

A) Schematic representation of the genomic locus of the TMEM16F gene (top), of the targeted allele (middle) and floxed and flipped alleles (bottom). *Neo* and *Kan*: Neomycin and Kanamycin resistance gene; *loxP* and *FRT* sites are indicated by grey arrowheads; restriction enzymes sites are indicated by blue arrowheads for *HindIII* and red arrowheads for *Kpn1*. B) Southern blot analysis of genomic DNA for indicated genotypes *TC*: targeted cells; fl/fl: floxed mice C) Representative western immunoblot of TMEM16F in three different tissues from indicated genotypes. Control: HEK293 cells transfected with TMEM16F DNA; liv: Liver homogenates; spl: spleen homogenates; br: brain homogenates; D) *TMEM16F* deletion influences the expected Mendelian ratios litters from $TMEM16F^{+/-}$ crosses (n=12 litters, $p<0.05$ χ^2 test) E) Representative Z-stack projection of the ipsilateral (top) and contralateral (bottom) dorsal horn of the spinal cord from a $CX3CR1^{cre}$::$Rosa26^{tdRFP}$ mouse 5 days after PNL. Anti-RFP (red) , in green anti-NeuN(green) staining. Scale bar: 50 µm.

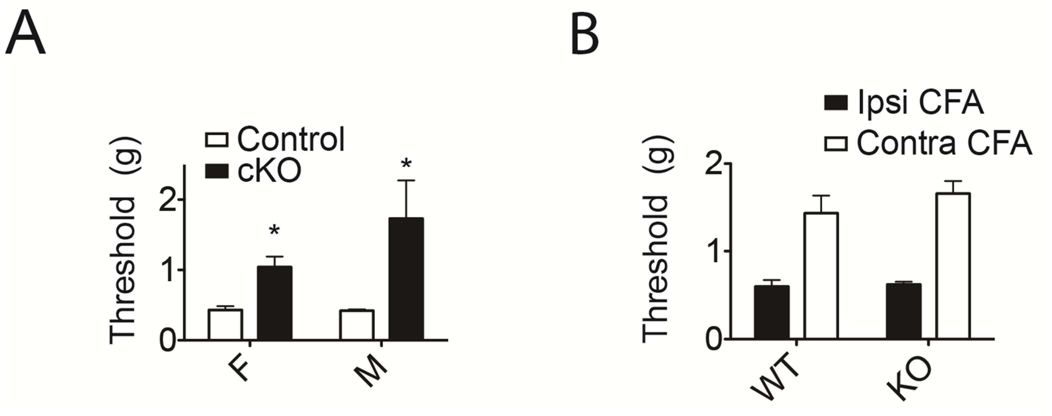

Figure S2, related to Fig. 2

(A) Graph showing the evoked responses to von Frey filaments of the ipsilateral (ipsi) and contralateral (contra) paw in female (F) and male (M) mice seven days after PNL. Data are expressed as paw withdrawal threshold value and are presented as mean ± SEM. N=4/5 for males and 4/3 for females; p<0.05 between genotypes, using One-Way ANOVA.

(B) Graph showing the evoked responses to von Frey filaments of the ipsilateral (ipsi) and contralateral (contra) paw two days after Complete Freunds Adjuvant (CFA) injection. Data are expressed as paw withdrawal threshold value and are presented as mean ± SEM. N=6/8; p>0.05 between genotypes, using One-Way ANOVA.

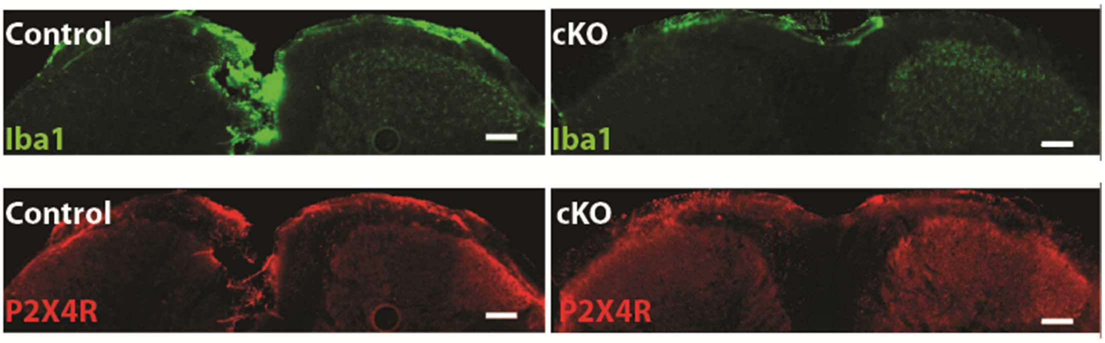

Figure S3, related to Fig. 2

Representative images of lumbar spinal cord slice from TMEM16F$^{fl/fl}$ (control) and *LysM^{cre}::TMEM16F$^{fl/fl}$* (cKO) mice stained with anti-Iba1 (green, top) and anti-P2X4 receptor (red, bottom) antibodies. P2X4 immunoreactivity is increased on the right side of the slice, ipsilateral to PNL, marked by numerous Iba1+ microglia. Scale bar 100 µm. Mean grey levels of P2X4 channel were measured across dorsal horn area (lamina I-III) and normalized to the contralateral side.

Movie S1, related to Fig. 4A

Representative time lapse movies showing fluorescent microglia branch motility towards an ATP pipette puff. *On the left*: *CX3CR1*^{GFP}*:: TMEM16f*$^{fl/fl}$ (Control^{GFP}).*On the right*: *CX3CR1*^{GFP}*::LysM*^{Cre}*::TMEM16f*$^{fl/fl}$ (cKO^{GFP}) mice.

T= 0 corresponds to start of acquisition; t=4 min corresponds to Mg-ATP application, 5 psi, 100 ms. Green squares indicate the point of the pipette tip.

Movie S2, related to Fig. 4E

Representative Z-stack time lapse movie showing segmented spinal microglia (green and yellow) from a Control^{GFP}mouse followed by a cKO^{GFP} mouse, and analysis of processes motility using a microglia tracking algorithm over time. One frame every minute for a total of 30 minutes. Scale bar 50 μm.

Movie S3 related to Fig. 4G

3D View of segmented GFP positive microglia (green) and segmented CTB-Alexa647 labelled neuronal terminals (cyan) in the dorsal horn of an ipsilateral spinal cord slice 3 days after surgery. Segmented neuronal terminals co-localized with GFP signal are considered internalized (purple).

Movie S4, related to Fig.4

Representative movie of microglia (blue) from *LysM*^{Cre}*::TMEM16F*$^{fl/+}$*::Rosa26*$^{Cl\text{-}Sensor}$ (Control$^{Cl\text{-}sensor}$) (left) and *LysM*^{Cre}*::TMEM16F*$^{fl/fl}$*::Rosa26*$^{Cl\text{-}Sensor}$ (cKO$^{Cl\text{-}Sensor}$) (right) mice, incubated with fluorescent yeast (orange) for a phagocytosis assay. One frame every minute for a total of 30 minutes.

Supplemental experimental procedures

Generation of the TMEM16F conditional knockout allele and genotyping

The TMEM16F targeting strategy was designed to allow Cre-mediated excision of the exons 13 of the TMEM16F, resulting in a frame-shift mutation in exon 14. The TMEM16F gene-targeting vector contained a loxP site located 5’ of exon 13, a neomycin resistance (neor) gene flanked with two frt sites, and a loxP site located 3’ of exon 13.

The targeting construct was transfected into A9 clone ES cells. Southern blotting of individual ES cell clones was used to identify homologous recombinants. DNA was digested with *Kpn1* or *HindIII* and hybridized with a 3` or 5` probe, respectively. Using the 5` probe, three DNA fragments were obtained: a 11600 bp (wild-type) fragment, a 7200 bp (targeted cells and floxed) fragments and 10900 bp (knock out) fragment. Using the 3` probe, two DNA fragments were obtained corresponding to a 9300 bp (wild-type) fragment, 7600 bp (targeted cells and floxed and knock-out) fragments. Positive clones were injected into 8-cell stage embryos to generate mice heterozygous for the targeted allele. Mice were first crossed with FLP-expressing transgenic mice to remove the frt flanked neoR cassette and then to $LysM^{Cre}$ or $Deleter^{Cre}$ mice. These genetic manipulations resulted in the generation of TMEM16F flipped and floxed alleles, TMEM16F conditional knockout alleles and TMEM16F null alleles, respectively. All analysis was performed on mice that did not carry the FLP or Del^{Cre} alleles.

Mouse genotype was verified by PCR using primers AAACAGTCACACTGGTGGGTGCC (forward for wild-type and LoxP (fl) allele), AGAGCAACTCCGCCAACCCCTTAG(reverse forward for wild-type and LoxP (fl) allele) and TAGACATTCTTTAGTGACAC (forward for null allele) and TGCTATGTTATCAAATACTAGC (reverse for null allele). The presence of the FLP transgene was determined with the following primers;

CCCATTCCATGCGGGGTATCG and GCATCTGGGAGATC ACTGAG. The presence of Cre recombinase was checked with primers GCACTGATTTCGACCAGGTT and GAGTCATCCTTAGCGCCGTA.All primers were purchased from Sigma Aldrich, Italy.

Animals

Mice were bred and maintained at the EMBL Mouse Biology Unit, Monterotondo, in accordance with Italian legislation under license from the Italian Ministry of Health.

TMEM16F conditional KO mice were crossed with the following mouse line: *CX3CR1*^{Cre}(Yona et al., 2013),*LysM*^{Cre} (Clausen et al., 1999), *Rosa26*^{tdRFP}(Luche et al., 2007), *Cl-sensor* (Batti et al., 2013), *CX3CR1*^{GFP} (Jung et al., 2000), Avil-*Cre::Rosa26*^{SNAPCaaX} (Yang et al., 2008).

LysM^{Cre}*::TMEM16F*$^{fl/fl}$ were referred to as cKO, *TMEM16F*$^{fl/fl}$ (in the absence of Cre) or *LysM*^{Cre}*::TMEM16F*$^{fl/+}$ mice were referred to as controls, *CX3CR1*^{GFP}*:: LysM*^{Cre}*::TMEM16F*$^{fl/-}$ and *CX3CR1*^{GFP}*::LysM*^{Cre}*::TMEM16F*$^{fl/fl}$ were referred to as cKO^{GFP}, *CX3CR1GFP::LysM*^{Cre}*::TMEM16F*$^{fl/+}$ or *CX3CR1*^{GFP}*::TMEM16F*$^{Fl/+}$ (in the absence of *Cre*) mice were referred to as Control^{GFP}, *Cre::TMEM16F*$^{fl/-}$*::Rosa26*$^{Cl-sensor}$ and *Cre::TMEM16F*$^{fl/fl}$*::Rosa26*$^{Cl-sensor}$ mice were referred to as cKO$^{Cl-sensor}$, *LysM*^{Cre}*::TMEM16F*$^{fl/+}$*::Rosa26*$^{Cl-Sensor}$ and *LysM*^{Cre}*::TMEM16F*$^{+/+}$*::Rosa26*$^{Cl-sensor}$ mice were referred to as Control$^{Cl-Sensor}$.

Pain models and intraneural Injection

Mice of both sexes were subjected to partial nerve ligation of the left sciatic nerve (Seltzer et al., 1990) under isoflurane anaesthesia (Esteve). Briefly, after exposure of the left sciatic nerve at high-thigh level a 7-0 silicon- treated silk suture (Ethicon, Italy) was inserted into the nerve and tightly ligated so that the dorsal 1/3-1/2 of the nerve thickness was trapped in the ligature. In sham operated mice the nerve was left intact and the wound was closed. In all animals, the right leg and sciatic nerve

were untouched; animals were tested three and seven days after surgery. Cholera Toxin-B (CTB) (lifetechnologies) injection was performed right after nerve ligation. The nerve was subjected to serial injection of CTB Alexa Fluor 647 conjugate (0.5%in 2 μl of saline solution).

Intraplantar injection of Complete Freunds Adjuvant (CFA) (Sigma Aldrich, Italy) was used to model inflammatory pain. 20 μl CFA (Heat-killed *Mycobacterium butyricum*,10 mg/ml in mineral oil) was injected into the left hind paw under brief isoflurane anaesthesia. The inflammation was confined to the left paw throughout the observation period. Sham animals were injected with 20 μl of mineral oil.

Behavioral analysis

All mice were habituated to the testing environment for three days. All mice were tested for mechanical hypersensitivity of the hindpaw one day before and three and seven days after surgery. For the inflammatory model animals were tested 48 hours after injection. Mechanical allodynia was measured using the von Frey test. Briefly, von Frey filaments ranging from 0.04 g to 1.2 g of force were applied perpendicularly to the plantar surface of the paw through wire-mesh observation cages. The paw withdrawal threshold (PWT) was determined by sequentially increasing the stimulus strength and fitting percentage withdrawal with sigmoidal function.

Cell culture preparation

Microglial cell cultures were obtained from mixed glia cultures derived from the cerebral cortices of newborn mice (P0-P2). Cortices were digested in 20 U/ml papain (Sigma Aldrich, Italy)for 20 min at 37 °C followed a gentle trituration. The dissociated cells were washed, suspended in DMEM (Lifetchnologies, Italy) supplemented with 10% fetal bovine serum (FBS, Lifetchnologies, Italy), 100 U/ml penicillin and 0.1 mg/ml streptomycin (Lifetchnologies, Italy), and plated (5×10^5cells/cm2) on flasks coated with poly-L-lysine (100 μg/ml) (Sigma Aldrich, Italy). After 7–9 days,

cells were shaken for 2 h at 37 °C to detach and collect microglial cells. These procedures gave almost pure microglial cell populations as previously described (Lauro et al., 2010). Once collected, microglial cells (10^5) were plated on round glass coverslips (12 mm diameter) or on glass bottom dishes (MatTek Corporation, USA) coated with poly-L-lysine (100 µg/ml) and used for experiments after 2-4 days.

Tissue slice preparation

Spinal cord slice were taken from 8-13 weeks old mice. After cervical dislocation a laminectomy was performed to expose the lumbar spinal cord. The L3-L5 segment of spinal cord was freed, embedded on 2% low melting agarose (Promega, USA) in water and incubated in cold HEPES buffer solution of the following composition (in mM): NaCl 140; 193 NaOH 4.55; HEPES 10; KCl 4; Glucose 5; $MgCl_2$ 1; $CaCl_2$ 2. pH 7.4. 120µm transverse free floating sections of L3-L5 spinal cord segment were obtained using Vibratome (Leica VT1000S) and left in HEPES buffer solution to recover for 30 minutes. Slice were then incubated in an imaging chamber and held in place with a U-shaped stainless steel rod. Imaging was carried out excluding the areas damaged by tissue sectioning 30 µm above and beneath the section.

Hippocampal slices were prepared from P30–P40 mice. Animals were decapitated after being anesthetized with halothane, and whole brains were rapidly immersed for 10 min in ice-cold continuously oxygenated (95% O2, 5% CO2; pH 7.4) artificial cerebrospinal fluid (ACSF) containing 125 mM NaCl, 2.5 mM KCl, 1 mM MgCl2, 2 mM CaCl2, 1.125 mM NaH2PO4, 10 mM glucose and 26 mM NaHCO3. Transverse 250-µm hippocampal slices were cut at 4 °C with a vibratome (DSK, Kyoto, Japan) in oxygenated ACSF and allowed to recover for 1 h in oxygenated 10% diluted ACSF (Hypo-ACSF, 270 mOsm). Individual slices were transferred to the recording chamber under the microscope and superfused with oxygenated Hypo-ACSF at the rate of 1.5 ml per min at room temperature (23–25 °C).

Confocal time-lapse microscopy

Time-lapse video microscopy was carried out on a spinning disk confocal ultraviewVox (Cellular imaging, Perkin Elmer) and diode solid lasers (wavelengths: 405, 488, 568 and 647nm) were used as excitation sources. Imaging was performed using a Hamamatsu EMCCD camera (Hamamatsu, Japan). The z-position was controlled by a nano-scan piezo-drive stage (Prior) via the Perkin-Elmer pilot software (VolocityImprovision, UK). Time-lapses were carried out at 37° C and 5%CO_2, unless is specified on the text.

For phagocytosis assays microglia cells expressing Cl-Sensor probe (Batti et al., 2013) were co-incubated with fluorescent heat killed *S. cerevisiae* yeast (2.5 x10^6 cells per dish). Images were taken on a Perkin Elmer spinning disk microscope every minute for 30 minutes.

For spinal cord *ex vivo* time lapse *Z* optical series covered 40 µm of thickness throughout the tissue with 0.5 µm thickness. Images were taken of the dorsal horn of the ipsilateral side of the spinal cord every minute for 30 minutes.

Wide-field time lapse imaging of microglial processes

Wide-field time-lapse fluorescence images in hippocampal slices were acquired at room temperature (24 - 25 °C) using a customized digital imaging microscope. Excitation of GFP was achieved using a 1-nm-bandwidth polychromatic light selector (Till Polychrome V), equipped with a 150 W xenon lamp (Till Photonics, Germany). Fluorescence was visualized using an upright microscope (Axioscope) equipped with a 40x water-immersion objective (AchroplanCarlZeiss, USA) and a digital 12 bit CCD camera system (SensiCam, PCO AG, Germany). All the peripheral hardware control, image acquisition and image processing were achieved using customized software Till Vision v. 4.0 (Till Photonics, Germany). A glass pipette containing adenosine 5'-triphosphate magnesium salt (ATP, 2 mM; Sigma Aldrich) was placed in the stratum radiatum in the center of the recording field. Mg-ATP was pressure applied to the

slices (100 ms; 5 psi) with a Picospritzer III (Parker Instrumentation). Changes in GFP fluorescence distribution was monitored by acquiring a fluorescent stack every 10 seconds for 50 minutes. To quantify the speed of microglial process rearrangement toward the pipette tip, the increase of GFP fluorescence was measured in a circular area centered on the pipette tip (10 µm radius). At each time point the fluorescence increase in the area was calculated as $\Delta F = F\text{-}F_0$, and then divided for F_0 ($\Delta F/F_0$, where F_0 is the average fluorescence before ATP puff) to normalize the difference in basal GFP fluorescence. Slices were used from 2 to 7 hours after cutting. For these experiments *CX3CR1*^{GFP}*:: LysM*^{Cre}*::TMEM16F*$^{fl/-}$ and *CX3CR1*^{GFP}*::LysM*^{Cre}*::TMEM16F*$^{fl/}$*fl* were used as cKO^{GFP} and *CX3CR1*^{GFP}*::TMEM16F*$^{Fl/+}$ and *CX3CR1*^{GFP}*::TMEM16F*$^{Fl/fl}$ (in the absence of Cre) mice were used as Control^{GFP}.

Tracking analysis of single microglial process

All images were processed using ImageJ software. Images stacks were exported as .avi files to enable manual cell processes tracking on the ImageJ “Manual Tracking” plug-in (http://imagej.nih.gov/ij/plugins/track/track.html). To obtain quantitative analysis of tracks parameters, data were analyzed with ImageJ and Origin 7 (OriginLab Co.) software. Stacks were first background subtracted to optimize contrast. To obtain x-y coordinates of single processes, track positions were transferred into a new coordinate system, in which the ATP-containing pipette tip was set as origin (x = 0, y = 0). For each moving process (i), with position vector Ri(t), the change in position from one frame to the next (ΔRi(t)), and the instantaneous velocity (vi(t)) were given by ΔRi(t) = Ri(t+Δt)− Ri(t), and vi(t) = ΔRi(t)/Δt respectively, where Δt is the elapsed time among the two frames. The mean elongation velocity of each process was calculated as <v>=dx/dt, expressed in µm/min, defining dx as the mean accumulated distance of each process i sampled within the time interval dt.

In Situ Hybridization

Cells grown on coverslips were fixed in 4% PFA for 30 min, washed in PBS and permeabilized by overnight incubation in 70% ethanol at 4C. In situ hybridization was carried out using an in vitro transcribed probe generated from the full coding region of TMEM 6F. Cells were acetylated and hybridized with the probe in 50% formamide, 5X SSC, 5x Denhardt's solution, 500 ug/ml salmon sperm DNA, and 250 ug/ml tRNA overnight at 56 C. After post-hybridization washes with 50% formamide, 2X SSC at 52.5 C, and with 2X SSC at ambient temperature, sections were blocked and incubated overnight with anti-digoxigenin-AP (Roche; at 1:1000). Signal detection was done using NBT/BCIP substrate.

RNA fluorescence in situ hybridization and immunofluorescence

Spinal cords were collected, fresh frozen in OCT and sectioned at 20 µm onto Superfrost Plus slides. In situ hybridization was performed using a Fluorescein-labeled probe generated from a full-length TMEM16F cDNA. Briefly, sections were fixed in 4% paraformaldehyde, digested with proteinase K for 5 min, acetylated, and hybridized with the probes in 50% formamide, 5X SSC, 5x Denhardt's solution, 500 ug/ml salmon sperm DNA, and 250 ug/ml tRNA overnight at 56º C. After post-hybridization washes with 50% formamide, 2X SSC at 45 C, and with 2X SSC at ambient temperature, sections were blocked and incubated overnight with mouse anti-fluorescein (Roche; at 1:100) and was detected by a donkey anti-mouse Alexa 555. The sections were then incubated with rabbit anti-Iba1 (Wako; at 1:200), followed by donkey anti-rabbit Alexa 488.

Real Time PCR

Homogenates of DRGs, Brain, Kidney, L4–L6 segment of the lumbar spinal cord and lysates of microglia cells were subjected to total RNA extraction with an RNeasy Micro Kit (Qiagen) according to the manufacturer's instruction. mRNA was reverse

transcribed to cDNA by Superscript II reverse transcription (Life Technology), using Oligo(dT) primers. Quantitative PCR was performed on a LightCycler 480 PCR instrument (Roche) using SYBR green I master (Roche). The primers were as follows: TMEM16A primers (forward: 5’-GAGGCCAGTAGCCATCAGAG-3’), (reverse: 5’-TCTGGAAGTCGCTGACATTG-3’), TMEM16B (forward: 5’-CGGATATCCCCACTGACATC-3’), (reverse: 5’-ACCCTGAGGATGCTATGCTG-3’) TMEM16C (forward: 5’-CGAAAGCCAAGTAAGCCAAG-3’), (reverse: 5’-AAGACTGTGGCCCATATTGC-3’), TMEM16D (forward: 5’-CGACTTCATCCCTCGCTTAG-3’), (reverse: 5’-TAGGGTGCCAAAGAGTGAGG-3’), TMEM16E (forward: 5’-CCAGGAGTATGAGAGCAGCC-3’), (reverse: 5’-AATGATGGTTAGCTGGGTGG-3’), TMEM16F (forward: 5’-GCACTTGGAGCAAAGAGGTC-3’), (reverse: 5’-TGCTGTAGCTCAACGGTGTC-3’), TMEM16G (forward: 5’-AAGGTACTTCGGGGAGAAGG-3’), (reverse: 5’-AAGAACAGTCGGAGCACAGC-3’), TMEM16H (forward: 5’-CAAGGTTATGCTAGCCCTGC-3’), (reverse: 5’-TCTTTTAGGCGGTCCATGTC-3’), TMEM16I (forward: 5’-ACCTTGTGGAAATCCGTCTG-3’), (reverse: 5’-ACGGGCCATAGTGGTACTTG-3’), TMEM16J (forward: 5’-ACCTTGTGGAAATCCGTCTG-3’), (reverse: 5’-ACGGGCCATAGTGGTACTTG-3’) for RT-PCR of TMEM16 family members. All primers were purchased from Sigma Aldrich, Italy. Each mRNA expression level was normalized to ubiquitin or GADPH.

Western blotting

Harvested tissues were homogenized on ice in buffer containing 10 mM Hepes, 1mM EDTA, 250 mM sucrose and proteinase inhibitor using a Politron homogenizer. Homogenates were centrifuged at 1500 rpm for 5 min at 4 ºC. For the membrane fraction, 100 mM NaCO3 (pH 11.2) was then added to the supernatant and shaken for 45 min at 4 ºC. Lysates were incubated in sample buffer for 30 min at 37 ºC. Lysates from HEK293 cells expressing TMEM16F were used as positive control. 10

µg lysates were loaded for gel electrophoresis. Proteins were transferred to a nitrocellulose membrane, and incubated with rabbit anti-TMEM16F (HPA038958, Sigma) antibody in 0.1% Tween TBS + 5% dry nonfat milk, and HRP-linked donkey anti-rabbit IgG (NA934V, GE Healthcare) secondary antibody. Detection was performed using the ECL Western blot chemiluminescent reagent (RPN2106, GE Healthcare).

Immunohistochemistry

Mice were anesthetized with 2.5% Avertin and subjected to intracardiac perfusion with fresh 4% paraformaldehyde in PBS. Spinal cord and sciatic nerve were dissected and postfixed with 4% paraformaldehyde in PBS for 120 and 30 minutes, respectively. L3-L5 Lumbar segments of the spinal cord were embedded in 2% low melting agarose in water and 50µm transverse free-floating section were obtained using a Vibratome (Leica). Free floating section were permeabilized in 0.5% Triton-X in PBS for 1 hour at room temperature. Unspecific binding was blocked using a 1 hour incubation with 1% BSA in 0.5% Triton X-100. Nerves were cryoprotected in 30% sucrose PBS, and cut on a cryostat (18 µm; Leica). Cryosections were permeabilized using 0.2% Triton X-100 in TBS for 10 minutes and blocked with 5% normal goat serum in 0.1% Tween-20 TBS for 30 minutes at room temperature. The following primary antibodies were incubated overnight at 4°C at the described dilution: rabbit anti-Iba1 (019-19741, Wako; 2.5 µg/ml), rat anti-CD68 (Abd Biotech; 10 µg/ml), mouse anti-NeuN (1:250), rabbit anti-P2X4 receptor (ab82329, Abcam; 1:200), rabbit anti-RFP (600-401-379, Rockland; 5 µg/ml), rabbit anti-GABA (A2052, Sigma;1:2000). For co-staining with RFP or P2X4R, a mouse goat anti-Iba1 (Novus; 5 µg/ml) was used. Anti-rabbit-, anti-rat and anti-mouse Alexa-488, -546 or -647 secondary antibodies (2 µg/ml) (Life Technologies) were applied and incubated for 90 minutes at room temperature. Dapi (5 µg/ml) was applied for 20 minutes and washed by PBS. Sections were mounted on slides using Pro-long Gold (Life Technologies). Confocal microscopy was performed using a TCS-SP5 (Leica) Laser

Scanning System. For free floating slice imaging, *Z* optical series covered 42 μm of thickness throughout the tissue with 0.5 μm thickness. For imaging of cryosections *Z* optical series covered 12 μm of thickness throughout the tissue with 0.5 μm thickness. Low magnification spinal cord tiled images were acquired using wide-field microscope (Leica Microsystems).

Confocal Image analysis

Three-dimensional reconstructions of confocal stacks and surface rendering were generated using Imarisbitplane© Software (Bitplane, Zurich, Switzerland). *Surface*, *Filament* and *Spots*Imaris modules were used for segmentation and tracking of microglial cells, microglia processes and neuron terminals, respectively. For microglia branch motility analysis, the volume of rectangular cuboid containing single objects was measured for each timepoint, using IMARIS *Surface* module. For detection of internalized neuronal terminals by microglia, objects identified by the *spots* module were filtered by mean intensity with a thresholded green (microglia) intensity. Volocity 3D image analysis software (Perkin Elmer) was used for three-dimensional reconstructions and quantitative analysis of confocal stack of nerve sections.

Cytokine arrays

Mice at day 4 after PNL induction were trascardially perfused with PBS, and sciatic nerve fragments were excised and immediately frozen in liquid nitrogen. Samples were thawed, homogenized and protein extracted. Contralateral and ipsilateral sciatic nerve protein extracts were pooled together from 4 mice to produce 130 μg protein. Each sample was incubated with a separate pre-spotted membrane of 40 cytokines/chemokines and processed according to manufacturer's instructions (R&D Systems, mouse cytokine array panel A, no. ARY006). The signal was revealed with streptavidin-HRP-conjugated secondary antibody and chemiluminescence. Intensity

of selected dots was analyzed in ImageJ, and mean pixel density for each protein was calculated by subtracting background and averaging the duplicates.

Statistical Analysis

Data analysis was performed with PRISM (GraphPad), Origin 6.1 (OriginLab Co.) and SigmaPlot (SyStat). All data are reported as mean ± s.e.m. Statistical significance was determined by one-way ANOVA test followed by Bonferroni *post hoc* test in case of significance, two-way ANOVA, Student's t-test, χ^2 test, Kolmogorov-Smirnov or Mann Whitney test.

Batti, L., Mukhtarov, M., Audero, E., Ivanov, A., Paolicelli, R.C., Zurborg, S., Gross, C., Bregestovski, P., and Heppenstall, P.A. (2013). Transgenic mouse lines for non-invasive ratiometric monitoring of intracellular chloride. Frontiers in molecular neuroscience *6*, 11.

Clausen, B.E., Burkhardt, C., Reith, W., Renkawitz, R., and Forster, I. (1999). Conditional gene targeting in macrophages and granulocytes using LysMcre mice. Transgenic research *8*, 265-277.

Jung, S., Aliberti, J., Graemmel, P., Sunshine, M.J., Kreutzberg, G.W., Sher, A., and Littman, D.R. (2000). Analysis of fractalkine receptor CX(3)CR1 function by targeted deletion and green fluorescent protein reporter gene insertion. Molecular and cellular biology *20*, 4106-4114.

Lauro, C., Cipriani, R., Catalano, M., Trettel, F., Chece, G., Brusadin, V., Antonilli, L., van Rooijen, N., Eusebi, F., Fredholm, B.B.*, et al.* (2010). Adenosine A1 receptors and microglial cells mediate CX3CL1-induced protection of hippocampal neurons against Glu-induced death. Neuropsychopharmacology : official publication of the American College of Neuropsychopharmacology *35*, 1550-1559.

Luche, H., Weber, O., Nageswara Rao, T., Blum, C., and Fehling, H.J. (2007). Faithful activation of an extra-bright red fluorescent protein in "knock-in" Cre-reporter

mice ideally suited for lineage tracing studies. European journal of immunology *37*, 43-53.

Schindelin, J., Arganda-Carreras, I., Frise, E., Kaynig, V., Longair, M., Pietzsch, T., Preibisch, S., Rueden, C., Saalfeld, S., Schmid, B.*, et al.* (2012). Fiji: an open-source platform for biological-image analysis. Nature methods *9*, 676-682.

Schneider, C.A., Rasband, W.S., and Eliceiri, K.W. (2012). NIH Image to ImageJ: 25 years of image analysis. Nature methods *9*, 671-675.

Schwenk, F., Baron, U., and Rajewsky, K. (1995). A cre-transgenic mouse strain for the ubiquitous deletion of loxP-flanked gene segments including deletion in germ cells. Nucleic Acids Res 23, 5080-5081.

Seltzer, Z., Dubner, R., and Shir, Y. (1990). A novel behavioral model of neuropathic pain disorders produced in rats by partial sciatic nerve injury. Pain *43*, 205-218.

Suzuki, J., Umeda, M., Sims, P.J., and Nagata, S. (2010). Calcium-dependent phospholipid scrambling by TMEM16F. Nature *468*, 834-838.

Yang, Y.D., Cho, H., Koo, J.Y., Tak, M.H., Cho, Y., Shim, W.S., Park, S.P., Lee, J., Lee, B., Kim, B.M.*, et al.* (2008). TMEM16A confers receptor-activated calcium-dependent chloride conductance. Nature *455*, 1210-1215.

Yona, S., Kim, K.W., Wolf, Y., Mildner, A., Varol, D., Breker, M., Strauss-Ayali, D., Viukov, S., Guilliams, M., Misharin, A.*, et al.* (2013). Fate mapping reveals origins and dynamics of monocytes and tissue macrophages under homeostasis. Immunity *38*, 79-91.
